# CRISPR-Cas9 globin editing can induce megabase-scale copy-neutral losses of heterozygosity in hematopoietic cells

**DOI:** 10.1038/s41467-021-25190-6

**Published:** 2021-08-13

**Authors:** J. Boutin, J. Rosier, D. Cappellen, F. Prat, J. Toutain, P. Pennamen, J. Bouron, C. Rooryck, J. P. Merlio, I. Lamrissi-Garcia, G. Cullot, S. Amintas, V. Guyonnet-Duperat, C. Ged, J. M. Blouin, E. Richard, S. Dabernat, F. Moreau-Gaudry, A. Bedel

**Affiliations:** 1 Bordeaux University, Bordeaux, France; 2 INSERM U1035, Biotherapy of Genetic Diseases, Inflammatory disorders and Cancers, Bordeaux, France; 3 University Hospital Bordeaux, Biochemistry Laboratory, Bordeaux, France; 4 Laboratory of Excellence, Gr-Ex, Bordeaux, France; 5 Bordeaux University, MRGM INSERM U1211, CHU de Bordeaux, Service de Génétique Médicale, Bordeaux, France; 6 University Hospital Bordeaux, Tumor Biology and Tumor Bank Laboratory, Bordeaux, France; 7 INSERM U1053, Bordeaux Research in Translational Oncology, Bordeaux, France; 8 INSERM US 005—CNRS UMS 342-TBM-Core, Bordeaux University, Bordeaux, France

**Keywords:** Targeted gene repair, CRISPR-Cas9 genome editing

## Abstract

CRISPR-Cas9 is a promising technology for gene therapy. However, the ON-target genotoxicity of CRISPR-Cas9 nuclease due to DNA double-strand breaks has received little attention and is probably underestimated. Here we report that genome editing targeting globin genes induces megabase-scale losses of heterozygosity (LOH) from the globin CRISPR-Cas9 cut-site to the telomere (5.2 Mb). In established lines, CRISPR-Cas9 nuclease induces frequent terminal chromosome 11p truncations and rare copy-neutral LOH. In primary hematopoietic progenitor/stem cells, we detect 1.1% of clones (7/648) with acquired megabase LOH induced by CRISPR-Cas9. In-depth analysis by SNP-array reveals the presence of copy-neutral LOH. This leads to 11p15.5 partial uniparental disomy, comprising two Chr11p15.5 imprinting centers (*H19/IGF2:IG-DMR/IC1* and *KCNQ1OT1:TSS-DMR/IC2*) and impacting *H19* and *IGF2* expression. While this genotoxicity is a safety concern for CRISPR clinical trials, it is also an opportunity to model copy-neutral-LOH for genetic diseases and cancers.

## Abstract (web summary)

CRISPR-Cas9 generates double-strand breaks, which pose a threat to genome integrity. Here the authors show loss of heterozygosity in edited hematopoietic progenitor cells.

## Introduction

Jasin and colleagues^1^ provided evidences of engineered nucleases interest for the induction of double-strand break (DSB) in order to be applied to the treatment of human diseases. CRISPR-Cas9 has become a universal and powerful method for the precise genome editing of many organisms^2,3^ to model diseases, study genotype-phenotype relationships and improve gene therapy. The modification of human hematopoietic stem/progenitor cells (HSPCs) by CRISPR-Cas9 has rapidly led to clinical trials (clinical trial.gov: NCT03655678/NCT03745287). However, nuclease-mediated DSBs might present drawbacks as previously described with I-Sce1, ZFN and TALEN^4^. Unwanted OFF-target effects have been widely described. While DNA-DSBs can lead to undesirable small insertions/deletions (Indels) and even chromosomal rearrangements (translocations, inversions and deletions)^5–7^, guide-RNA (gRNA) design optimization and the use of highly specific Cas9 nucleases can control them. However, safety issues regarding genome ON-target integrity lack in-depth exploration. In addition to small Indels, a single ON-target DSB can lead to large deletions up to several kilobases in size, symmetrical or not, in mouse embryos^8,9^, in mouse hematopoietic progenitors, in human immortalized differentiated cells^10^ and human embryos^11^. Recently, we reported that CRISPR-Cas9 can even cause megabase-scale chromosomal truncations targeting Chromosome 10q (Chr10q) in two human cell lines and in human primary fibroblasts deficient for the tumor suppressor p53^12^. These large mono-allelic genomic deletions were confirmed in human colorectal carcinoma HCT116 cells targeting Chr18q arm terminal deletion^13^, in human primary iPSC targeting Chr7 and Chr21^14^, and very recently in early human embryos with risk of aneuploidy^14,15^. In addition, more complex rearrangements were present^8,10–12^, suggesting that the diversity of potential deletion outcomes is vast. These genomic alterations have not been reported until now because standard genotyping methods frequently miss them. Reassuringly, to date these genomic rearrangements have not been linked to deleterious consequences for patients.

It is currently unknown whether large alterations can occur after DSB in human HSPCs, the relevant clinical target cells for gene therapy protocols. Indeed, HSPCs benefit from superior p53-dependent capacity to prevent the accumulation of genetic lesions, repair them, and avoid their propagation to daughter cells^16–18^. In this work we observe that CRISPR-Cas9 nuclease induces frequent terminal chromosome 11p truncations in cell line and rare extra-large terminal copy-neutral loss-of-heterozygosity in HSPCs resulting in partial uniparental disomy of the 11p15.4-11p15.5 imprinted region altering *H19* and *IGF2* gene expression.

## Results

### Globin gene targeting induces extra-large Chr11p LOH by chromosomal terminal deletions in HEK cells

**Fig. 1:**
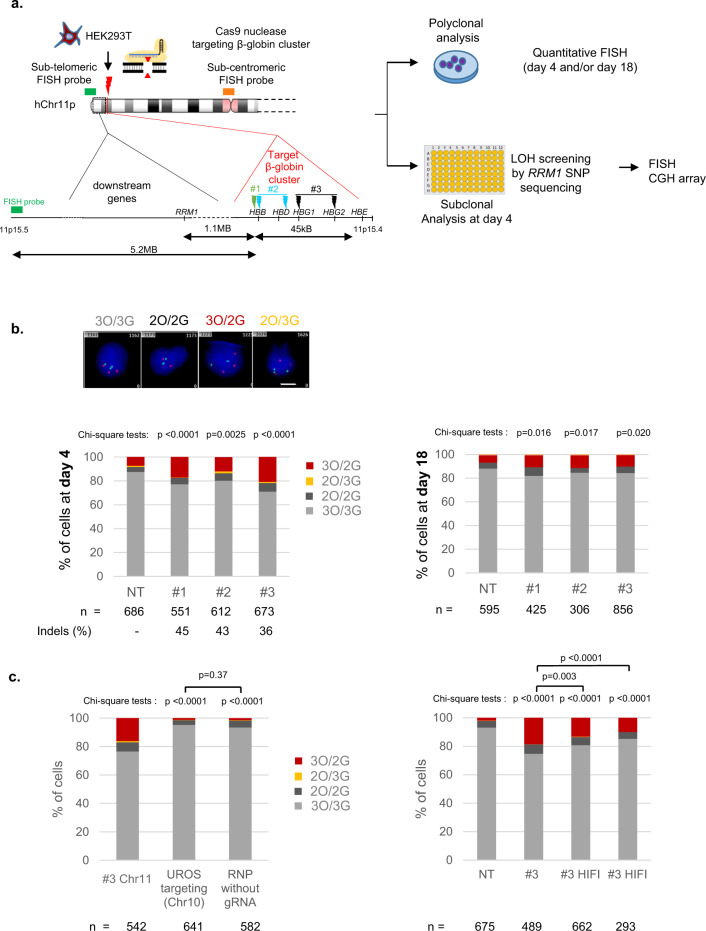
CRISPR-Cas9-mediated globin gene targeting induces Chr11p terminal deletions in HEK cells. **a** Experimental design. HEK293T cells edited with Cas9 nuclease RNP and sets of gRNA corresponding to protocols #1 to #3. Map of β-globin genes, telomeric downstream SNP genes (*RRM1)* and FISH probes. LOH, loss of heterozygosity; SNP, single-nucleotide polymorphism; FISH, fluorescent in situ hybridization; CGH, comparative genomic hybridization. **b** Illustrative Chr11 DNA-FISH in HEK cells. Scale-bar: 5 µm. Polyclonal Chr11 DNA-FISH analysis at D4 (left) and at D18 (right) after editing with protocols #1 to #3. NT: non-transfected. O, Orange sub-centromeric probe; G sub-telomeric Green probe. 3O/3G (in gray), 3O/2G (in red), 2O/3G (in yellow), and 2O/2G (in black) signal percentages are quantified. 3O/2G frequencies after protocols 1–3 were compared to non-transduced cells on same day by two-sided Chi-square tests. *n* correspond to analyzed cells from each polyclonal pool (1 per protocol). **c** left: Polyclonal Chr11 DNA-FISH analysis at D4 after editing with protocol #3 (Chr11), without gRNA or targeting another chromosome (*UROS* locus in Chr10). 3O/2G frequencies targeting Chr10 or without guide RNA were compared to protocol #3 on same day. 3O/2G frequencies targeting Chr10 or without guide RNA were compared by two-sided Chi-square tests. Right: Polyclonal Chr11 DNA-FISH analysis at D4 after editing with protocol #3 (Chr11), and with protocol #3 using HiFi Cas9. 3O/3G, 3O/3G, 2O/3G and 2O/2G signal percentages were quantified. 3O/2G frequencies with protocol #3 using HiFi Cas9 or Cas9 were compared to non-transduced cells by two-sided Chi-square tests. 3O/2G frequencies with protocol #3 using HiFi Cas9 were compared to protocol #3 using Cas9 by two-sided Chi-square tests. O: orange sub-centromeric Chr11 probe, G: green sub-telomeric Chr11p probe. n correspond to analyzed cells from each polyclonal pool (1 per protocol). Source data are provided as a Source Data file.

As we already reported truncations on Chr10q by targeting *UROS*^12^, we first evaluated whether such an event also occurs on Chr11p when targeting the globin genes^-^ in HEK293T cells. These cells are known to have low p53 activity, a favorable condition not only for editing^17,18^ but also for genomic instability. We designed three gRNA sets (Fig. 1a). The first one (protocol #1) targets the sixth codon of wild type *HBB*, which is mutated in sickle-cell disease (green lightning). The two other sets (protocols #2^19^ (blue lightning) and #3^20^ (black lightning)) are related to those previously published in successful preclinical studies for β-hemoglobinopathies gene therapy. CRISPR-Cas9 targeting by protocols #2 and #3 increases hemoglobin F (HbF) synthesis, recapitulating the hereditary persistence of fetal hemoglobin (HPFH)-associated mutations. Protocol #2 targets a 13-nt sequence present in the promoters of the *HBG1* and *HBG2* genes^19^. Protocol #3 uses 2 gRNAs to disrupt a 13.6 kb genomic region encompassing the δ- and β-globin genes and a putative γ-δ intergenic HbF silencer^20^ (Fig. 1a). We transfected each gRNA set with the ribonucleoprotein (RNP) Cas9 nuclease. After transfection, ICE (Inference of CRISPR Edits) analysis of the Sanger sequences confirmed the presence of a high rate of indels with all gRNAs (83%, 63%, and 60% for #1, #2 and #3, respectively). To evaluate Chr11p arm genomic integrity, we first performed fluorescent in situ hybridization (FISH) on HEK293T cells 4 days after editing (mainly trisomic for Chr11). We used a Chr11-specific orange sub-centromeric probe (O) and a Chr11p-specific sub-telomeric green probe (G) at 5.2 Mb from the globin cluster. Blind deep FISH analysis by an automated system revealed that the cells displaying a 3O/2G profile increased from 7.3% (before transfection) to 17.2%, 11.9%, 20.8% after protocols #1, #2 and #3, respectively (Fig. 1b day 4 and Supplementary Figs. 1–3, >550 cells per condition). At the same time, Chr11 2O/3G cell percentages and FISH analysis of another chromosome (Chr2) to monitor technical noise were low and stable (around 1% in all conditions) (Fig. 1b and Supplementary Fig. 4). Around 5% of cells were disomic for Chr11 in control cells and the prevalence was stable in edited cells, which is not in favor of induced loss of the entire chromosome. Finally, targeting another chromosome (Chr10) with nuclease did not impact 3O/2G Chr11-cell percentages, indicating that Chr11-specific sub-telomeric probe hybridization loss increases at day 4 were specific genetic outcomes due to Cas9 nuclease use in Chr11p **(**Fig. 1c, left). Regardless of the gRNAs and the protocols, higher frequencies of unbalance in Chr11p copies suggested that these events were likely independent of the location or the number of DSBs. Sub-telomeric green probe losses were less frequent in protocols #1 and #3 at D18, but still persisted even after numerous cell divisions (at around 10% in all conditions without significant difference between protocols, *p* = 0.838 two-sided Chi-square tests) and was significantly higher than in unedited cells (NT, non-transfected: 6.2%) (Fig. 1b right, day 18). In contrast, Chr2 DNA FISH at day 18 showed stability of this chromosome **(**Supplementary Fig. 4**)**. These sub-telomeric probe losses could be consistent with extra-large terminal deletions of Chr11 (5.2 Mb) downstream of the globin locus, induced by nuclease-mediated DSB.

**Fig. 2:**
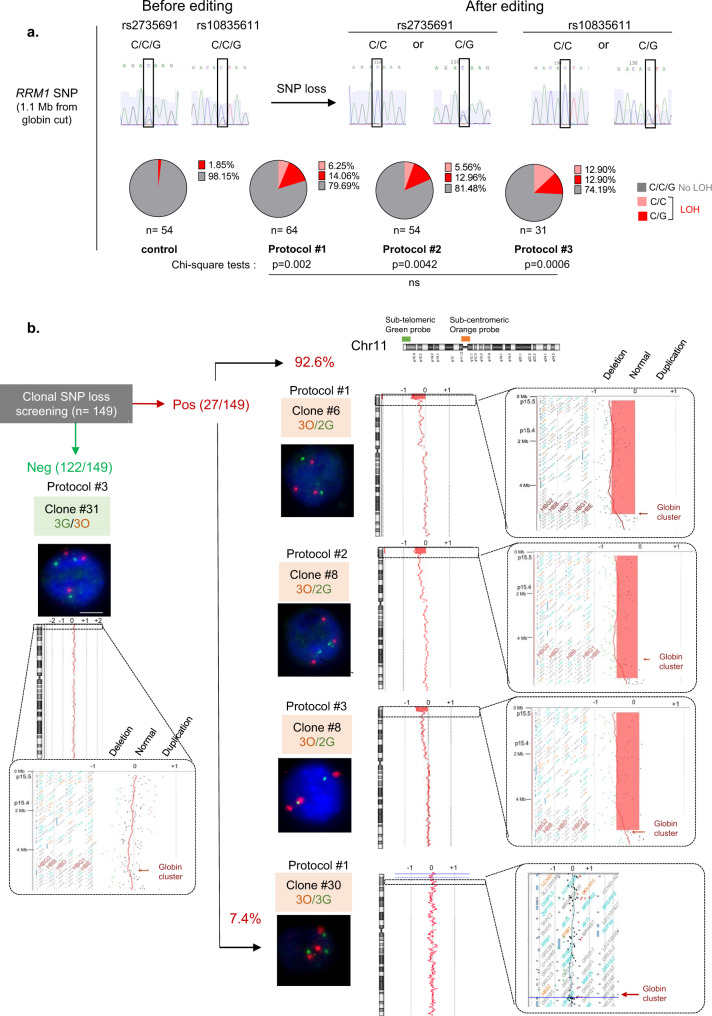
Confirmation and mapping of Chr11p terminal deletions. **a***RRM1/*SNP sequencing after subclonal analysis of HEK293T at D4. C/C/G genotype in *RRM1* (in gray) on two SNP positions for NT cells. After editing, clones presented SNP-LOH with C/C (in pink) or C/G genotype (in red). Frequencies of SNP losses are indicated in pie chart for each protocol. LOH frequencies with protocols 1–3 were compared to non-transduced cells by two-sided Chi-square tests. Indels frequency was 63, 83 56% for protocols 1–3, respectively, *n* correspond to analyzed cells from each polyclonal pool (1 per protocol). **b** Chromosome analysis in HEK293T clones depending on SNP screening. Left, DNA-FISH (Scale-bar: 5 µm) and array-CGH of one LOH-negative clone. Right, DNA-FISH and array-CGH of LOH-positive clones from protocols #1–3. Deleted area is in red. *n* correspond to analyzed SNP loss cells (from protocols #1–3). Source data are provided as a Source Data file.

To confirm this hypothesis and estimate the incidence of these outcomes, we carried out cellular subcloning and picked up 203 clones (54, 64, 54, 31 from control and protocols #1-2-3, respectively, Fig. 2a). To detect megabase deletions, we screened for homozygosity two single-nucleotide polymorphisms (SNP, rs2735691/rs10835611) in *RRM1*, heterozygous before editing and located 1.1 Mb telomeric from the Cas9 breakpoint. While only 1.9% (1/54) of cells lost *RRM1* SNP heterozygosity in the control cells, 20.3% (13/64), 18.5% (10/54) and 25.8% (8/31) of clones exhibited LOH in protocols #1 to #3, respectively, without significant difference between the protocols (Fig. 2a). 25/27 of the clones with *RRM1* SNP LOH (92.6%) screened by FISH lost one sub-telomeric green probe (3O/2G) in favor of a Chr11p terminal deletion (Fig. 2b and Supplementary Table 1). Array-CGH (Array Comparative Genomic Hybridization) identified the location of the globin genes targeted by CRISPR-Cas9 as the start of the breakpoint delimiting this 5.2-megabase terminal deletion in HEK293T cells and confirmed these terminal deletions, whatever the protocol used (Fig. 2b). Interestingly, 2/27 clones (7.4% of *RRM1* LOH clones) kept 3O/3G signals, suggesting a copy-neutral LOH by mitotic recombination confirmed by CGH-array (Fig. 2b and Supplementary Table 1**)**.

To conclude, in HEK293T cells, CRISPR-Cas9 nuclease induced frequent terminal Chr11p truncations and rare copy-neutral LOH.

None of the top-10-predicted off-target sites were situated on Chr11 (Supplementary Table 2), suggesting that these genetic outcomes were not due to additional off-target cut in the Chr11p. To clarify this prediction, we used HiFi Cas9, which is known to drastically reduce off-target cuts. HiFi Cas9 also increased the frequency of loss of the sub-telomeric probe, confirming that these chromosomal rearrangements were not caused by off-target effects **(**Fig. 1c right**)**. Frequency of 3G/2O cells with HiFi was twofold lower compared to protocol #3 using regular Cas9 but with a cut-off efficiency also reduced by half (in our experiment, 31% of indels with HiFi versus 61% with wild type Cas9). Taken together these data demonstrate that induced LOH are not dependent to off-target activity of Cas9 nuclease.

### CRISPR-Cas9-mediated globin gene targeting can induce extra-large Chr11p CN-LOH in human hematopoietic stem/progenitor cells (HSPCs)

**Fig. 3:**
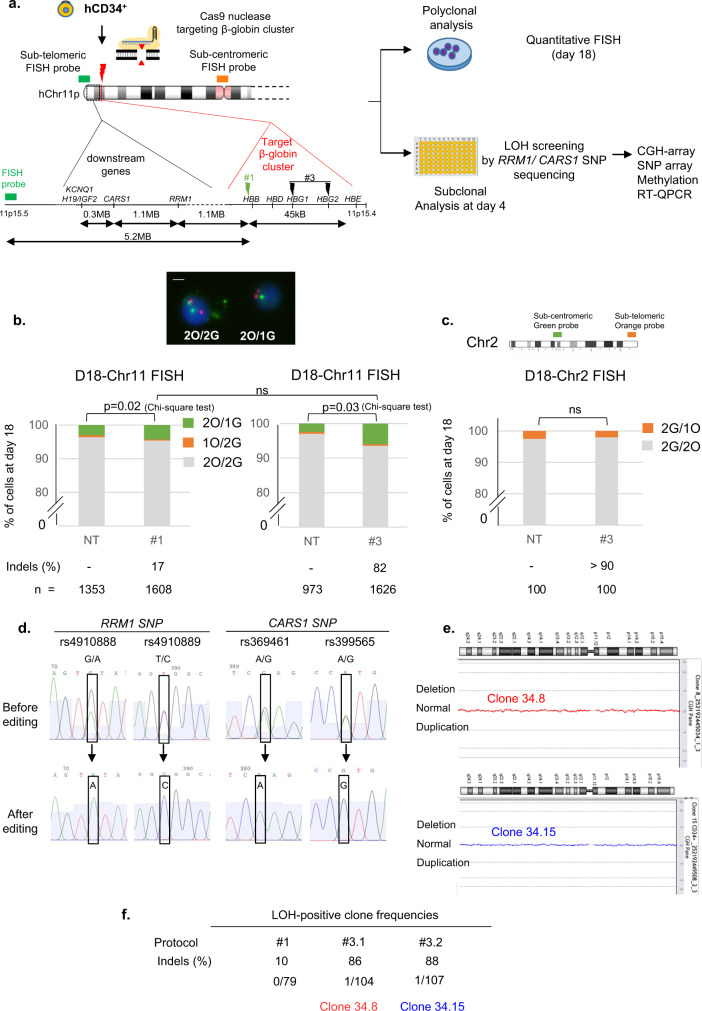
CRISPR-Cas9-mediated globin gene targeting can induce Chr11p copy-neutral-LOH (iCN-LOH) in HSPC. **a** Experimental design human CD34+ from cord blood edited with Cas9 RNP and sets of gRNA corresponding to protocols #1 and #3. Map of β-globin genes, telomeric downstream SNP genes (*RRM1, CARS1 and KCNQ1)*, telomeric imprinted genes (*H19* and *IGF2*) and FISH probes. **b** Top panel illustrative Chr11 DNA-FISH in hCD34^+^ cells. Scale-bar: 5 µm. Bottom panel, polyclonal Chr11 DNA-FISH analysis at D18 of edited cells with protocols #1 (left) and #3 (right). Median of each Chr11 profile (2O/2G (in gray), 2O/1G (in green), 1O/2G (in orange)) in non-transfected cells as control (NT, Non-Transfected) and edited cells with protocols #1 and #3 (*n* = 7). O: orange sub-centromeric Chr11 probe, G: green sub-telomeric Chr11p probe. 2O/1G frequencies were compared between conditions by two-sided Chi-square tests. n correspond to analyzed hCD34^+^ cells for each protocol (pooled from three distinct transfections). **c** Polyclonal Chr2 DNA-FISH analysis at day 18 of edited cells (in Chr11) with protocol #3 or non-transfected cells. O: orange sub-telomeric Chr2 probe, G: green sub-centromeric Chr2 probe. 2G/1O (in orange) frequencies were compared between conditions by two-sided Chi-square tests. *n* correspond to analyzed hCD34^+^ cells for each protocol. **d** *RRM1/CARS1* SNPs sequencing after subclonal analysis of CD34+ cells at D4, illustrating SNP-LOH observed in clones 34.8 and 34.15 from protocols #3.1 and #3.2. **e** Chromosome analysis of two HSPC clones with LOH by array-CGH (34.8 and 34.15). **f** LOH-positive clone frequencies for protocols #1, #3.1 and #3.2. Source data are provided as a Source Data file.

**Fig. 4:**
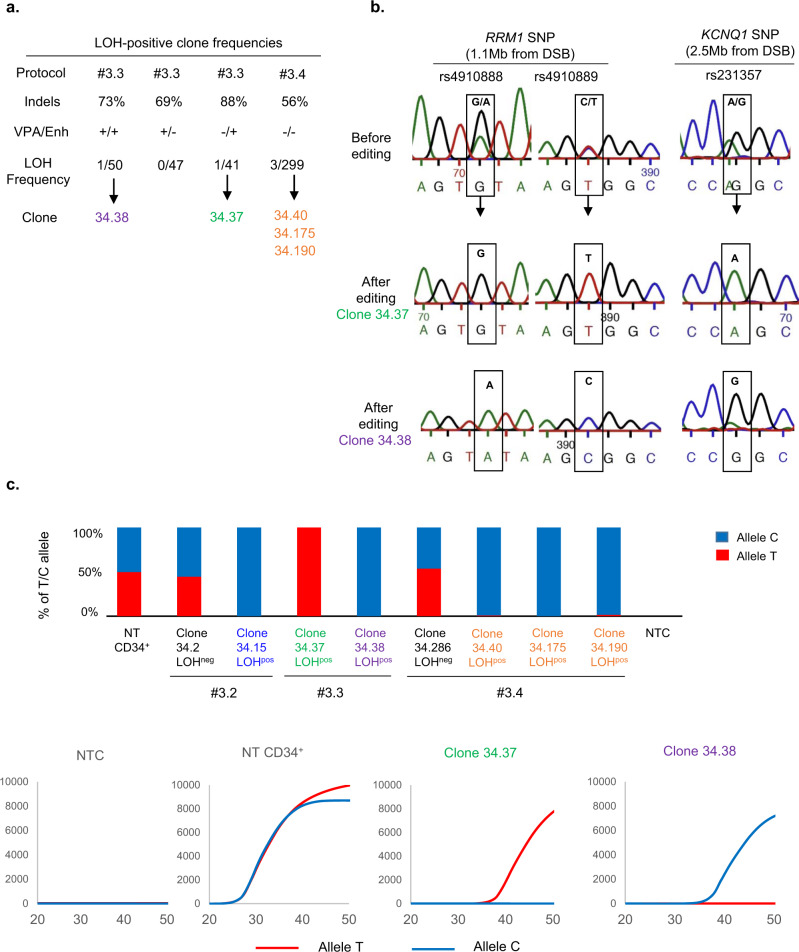
CD34+ LOH clone presence is not dependent of VPA and enhancer. **a** LOH-positive clone frequencies in experiments with or without valproic acid (VPA) and transfection enhancer (enh). **b** Illustrative Sanger sequencing of 2 *RRM1* SNPs at 1.1 Mb from the cut-site (rs4910888 and rs4910889) and *KCNQ1* SNP at 2.5 Mb from the cut-site (rs231357). **c** Allele-specific qPCR of *RRM1* (rs4910889) to determine presence or absence of SNP with illustrative curves (low panel). Non-transduced CD34+ (NT), clone 34.2 and 34.286 without LOH are heterozygous with T/C alleles (allele C in blue and allele T in red). Presence of single allele revealed LOH in numerous clones. NTC, No template control. Source data are provided as a Source Data file.

We then investigated whether these putative side-effects occurred in the clinically relevant HSPCs using CD34^+^ cord-blood cells. We repeated protocols #1 and #3 in diploid HSPCs by inducing one or two cuts, respectively (Fig. 3a). FISH analyses at D18 revealed slight increases in 2O/1G cells with both protocols in both experiments (+1.3% and +3.55% for #1 and #3, respectively, versus non-transfected (NT) cells, without a significant difference between the two protocols Fig. 3b, Supplementary Table 3, Supplementary Figs. 5 and 6). Chr11 1O/2G and Chr2 2G/1O cell percentages (Fig. 3b, c) did not increase. On the contrary, green probe losses (2O/1G) were suggestive of 11p terminal deletions, but the high background noise of 2O/1G in non-transduced cells makes the analysis complicated and international cytogenetic guidelines consider that the increase must be greater than 5% to be considered significant^21^. To improve sensitivity and conclude, we subcloned HSPCs by FACS cell sorting at day 4 post-editing. We first screened LOH in CD34^+^ clones by analyzing 4 SNPs in *RRM1* and *CARS1 loci*, 1.1 and 2.2 Mb telomeric to the globin cluster. We did not find any SNP loss in subclones after protocol #1 but we were able to analyze only 79 clones and guide RNA activity was low (indel rate: 10%). As LOHs seem to be DSB frequency-dependent, we chose protocol #3 for extensive cloning experiments. We performed two independent experiments of protocol #3 (indel rates of 86 and 88%), revealing the presence of two clones that lost one allele of the four SNPs (1/104, 1/107, in #3.1, #3.2, respectively, Fig. 3d, f). To be sure that these results were not due to the use of valproic acid (VPA, which improves CD34^+^ cell subcloning) and AMAXA nucleofector enhancer (enh, recommended by the manufacturer to increase editing efficiency), the same experiments were performed without VPA (VPA−/enh+) or the enh (VPA+/enh−). Clonal analysis revealed the presence of one clone with *RRM1* SNPs losses (at 1.1 Mb from the cut-site) and *KCNQ1* SNP loss (at 2.5 Mb from the cut-site), in the VPA−/enh+ condition (1/41 clones). The frequency of LOH was similar to that of the control VPA+/enh+ (1/50 clones) (Fig. 4a, b, c). We did not find any LOH clones in VPA+/enh− condition (0/47 clones). Therefore, we removed both VPA and enh and increased the number of analyzed clones to establish whether the presence of LOH was dependent on enh. We obtained 3/299 colony-forming cells (CFC) with LOH including *RRM1* and *KCNQ1* loci (Fig. 4a, c), confirming the absence of VPA and enh bias. Taken together, in primary hematopoietic progenitor/stem cells, we detected 1.1% of clones (7/648) with acquired megabase LOH induced by CRISPR-Cas9.

**Fig. 5:**
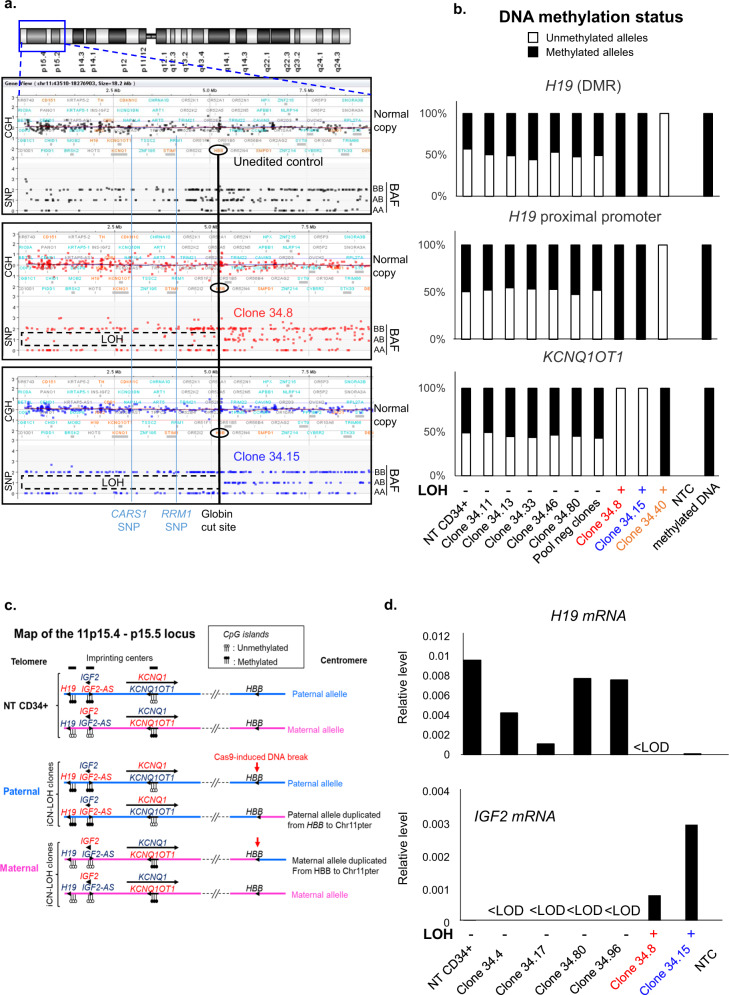
CRISPR-Cas9-Induced Chr11p copy-neutral-LOH (iCN-LOH) impacts methylation of imprinting centers and imprinted genes expression in HSPCs. **a** CGH/SNP-array: Log_2_Ratio and BAF (B-allele frequency) in control-HSPC cells and two edited LOH-positive-clones, zoomed on Chr11p15. Globin cut-site location is indicated by a black line. *RRM1* and *CARS* SNP losses are indicated by a blue line. LOH are framed. **b** Percentages of unmethylated and methylated alleles of *H19* differentially methylated region (*H19 DMR*), *H19* gene proximal promoter and *KCNQ1OT1* (*KCNQ1* opposite strand/antisense transcript 1) in NT-CD34+ (non-transduced parental CD34+ cells), in five edited clones without LOH (34.11, 34.13, 34.33, 34.36, 34.80), in a pool of negative LOH edited clones, in 3 clones with LOH (34.8, 34.15 and 34.40) analyzed by quantitative real-time methylation-specific PCR (qMSP). in vitro methylated DNA and No DNA (NTC) were used as positive and negative controls. ND: non determined because of lack of DNA cell material. **c** Diagram of Chr11p15.4-p15.5 band with position of imprinted genes and CRISPR-Cas9-induced DNA-DSB (red arrow) in HSPCs. Paternal and maternal alleles in light blue and pink, respectively. Unmethylated and methylated CpG islands represented by white and black circles. Genes expressed from parental allele in dark blue, gene silenced on parental allele in red. Normal paternal and maternal allele from NT-CD34+ cells and alleles from paternal and maternal iCN-LOH clones are represented. **d** *H19* and *IGF2* mRNA expression in NT-CD34+ cells, in four edited LOH-negative clones (34.4, 34.17, 34.80, and 34.96) and two LOH-positive clones (34.8 and 34.15). Results are normalized using TATA-box binding protein (*TBP*) housekeeping gene. LOD, limit of detection. Quantification is represented by mean and SEM from the same sample measured repeatedly. *H19* mRNA is not or hardly detectable in LOH-positive clones. *IGF2* mRNA is only detectable in LOH-positive clones. Source data are provided as a Source Data file.

For two clones (34.8 and 34.15) we have the chance to have enough material to explore in-depth these LOH induced by CRISPR-Cas9. Unexpectedly, array-CGH did not show any loss of genomic material (Fig. 3e), suggesting that these megabase-scale LOH were not due to terminal chromosomal deletions but to the occurrence of a copy-neutral loss of heterozygosity (CN-LOH), also called partial uniparental disomy. CGH/SNP-array analysis validated this hypothesis and mapped a single specific induced CN-LOH (iCN-LOH) in both clones spanning 5.2 Mb from the globin gene cut to the telomere (Fig. 5a and Supplementary Fig 7**)**. Sanger sequencing of SNPs along the Chr11p arm again confirmed these unexpected extra-large CN-LOH in primary hematopoietic cells probably due to mitotic recombination (Supplementary Fig. 8). To more precisely map the start of LOH, we carried out Sanger sequencing of the breakpoint of the two clones. Clone 34.15 analysis revealed a homozygous 4-base pairs deletion, suggesting that the CN-LOH starts exactly at the CRISPR-Cas9-cut-site. Two different edited alleles were present in clone 34.8, suggesting that CN-LOH starts between the cut-site and the first probe of the SNP array at 0.4 Mb (Supplementary Fig. 9a). We chose additional SNPs nearer to the CRISPR cut-site and sequenced them. We found an additional SNP (not studied by SNP array) located 0.18 Mb from the cut-site on the telomeric side. It became homozygous in the two CD34 clones, demonstrating that the LOH is already present. As expected, the SNP on the centromeric side (0.06 Mb from the globin cut-site) were still heterozygous (supplementary Fig. 9b). Taken together, these data highlight the presence of CN-LOH induced by CRISPR-Cas9 in HSPC starting in the surroundings of the DSB.

### Functional consequences of iCN-LOH

As the *11p15.4-11p15.5* locus is subjected to parental genomic imprinting^22^, we analyzed the methylation status of *H19* (a non-coding RNA tumor suppressor with maternal expression), *IGF2-AS* (insulin-like growth factor II gene, pro-oncogenic gene expressed only from the paternal allele) and the *KCNQ1OT1* imprinting centers in three LOH-positive clones compared to five LOH-negative clones. Normal cells harbor both unmethylated and methylated alleles of the *H19, IGF2-AS and KCNQ1OT1* regions from paternal and maternal alleles (Fig. 5b, c and Supplementary Fig. 10). Some HSPC clones (34.8 and 34.15) with CN-LOH exhibited bi-allelic methylation of the *H19* Differentially Methylated Region (DMR)/proximal promoter and the *IGF2-AS* DMR imprinting centers, and a bi-allelically unmethylated *KCN1QOT1* locus, indicative of partial paternal uniparental disomy impacting these imprinting centers. One other HSPC LOH-clone (34.40) showed an opposite imprinting profile indicative a partial maternal uniparental disomy showing that both profiles are possible without parental bias in vitro (Fig. 5b, c and Supplementary Fig. 10). Taken together, based on the haplotype, we obtained seven clones including three paternal duplications (34.8, 34.15, and 34.37) and four maternal duplications (34.38; 34.40; 34.175 and 34.190). We then evaluated the transcriptional modifications due to abnormal methylations in two LOH clones with partial paternal uniparental disomy (34.8 and 34.15) and four negatives clones. *H19* expression analysis of both 34.15 and 34.8 clones revealed silencing of the *H19* gene, consistent with the paternal uniparental disomy (UPD) of this locus and complete methylation of the gene in these cells. On the contrary, methylation of *IGF2-AS* resulted in the variable but significant slight induction of *IGF2* compared to negative clones (Fig. 5d).

## Discussion

In this study, we focused on the extra-large ON-target events that may occur when using CRISPR-Cas9 nuclease in the quest to establish appropriate quality-control measures when editing primary and animal cells. We report an ON-target genotoxicity of CRISPR-Cas9 nuclease in HSPCs. In these clinically relevant cells, we discovered that DNA DSB can lead in vitro to megabase-scale iCN-LOH. We subcloned HSPSCs from four independent transfections with protocol #3. We found 1.1% of clones (7/648) with acquired megabase LOH induced by CRISPR-Cas9, suggesting that these events are not so rare, considering that millions of edited cells needed to be transplanted. While no loss of genetic material occurs, these events should not be underestimated. Indeed, they may reveal recessive mutations. Cancer cells frequently acquire a growth advantage through CN-LOH by duplicating activating mutations in oncogenes or loosing wild-type alleles of tumor suppressor genes in many cancers^23,24^ including leukemia^25^.

Recent literature suggests that this process could be locus and cell type-independent. Davis et al.^26^ recently described CN-LOH due to CRISPR-Cas9-mediated DSB in cancer cell lines targeting Chr11 but at another locus 11p13 (CD44). Importantly, these events were also observed in human-induced pluripotent stem cells (iPSC) targeting Chr7 and Chr21^14^ and are still debated in human embryos^15,27^. Obviously, the consequences will likely be locus-dependent.

Here, we observed a 5.2 Mb iCN-LOH comprising 167 genes, notably the imprinted *H19* and *CDKN1C (*cyclin dependent kinase inhibitor 1C*)* tumor suppressors and the *IGF2* oncogene. We found that HSPCs with iCN-LOH harbor a bi-allelic paternal methylation pattern encompassing the imprinted *11p15.4-11p15.5* tumor suppressor domain, which contains the maternally expressed *CDKN1C* and *H19* and the paternally expressed pro-oncogenic *IGF2*. CN-LOH-associated disomy of the methylated or unmethylated alleles led to either complete inactivation or enhanced expression of the implicated genes. Among the seven LOH clones, three showed paternal isodisomy. The presence of a paternal profile could be deleterious, with *H19* silencing and *IGF2* slight induction. Single-cell RNA-seq analysis in thousands of clones will be informative to confirm transcriptional deregulation of *H19* and *IGF2*. Indeed, paternal isodisomy is a mechanism that is known to predispose to tumors in Beckwith-Wiedemann syndrome^28^. Spontaneous LOH occurs unfrequently in human cells (<0.01%)^26^. The high frequency of in vitro LOH starting at the cut-site described in this work cannot be the result of chance. Supplementary in-vivo experiments (graft in immunodeficient mice) will be required to evaluate (i) the graft capability of these rearranged cells, (ii) the long-term phenotypic consequences, and (iii) the putative selective advantage of paternal clones.

Targeting *BCL11A* for the induction of fetal hemoglobin^29^ is an alternative therapeutic approach for hemoglobinopathies. Its genotoxicity will need to be evaluated and compared to globin targeting. Interestingly, there is no imprinting center downstream of the *BCL11A* cleavage site and it could be safer. More generally, in light of our results, it is important to study the cancer predisposition genes distal to the DSB, notably the tumor suppressor genes whose heterozygous inactivating mutations would initiate or promote oncogenesis upon LOH targeting the functional allele.

We also demonstrate here that globin gene editing can induce frequent extra-large chromosomal deletions and rare CN-LOH in HEK cells. Our result are in accordance with previous publications reporting the risk of chromosomal truncation in cancer cell lines^12,13^. Altogether, these studies highlight a non-locus-dependent risk. Such large-scale rearrangements with CRISPR-Cas9 were recently described in human iPSC^14^ and human embryos^15^. We did not succeed in isolating truncated HSPC clones. We cannot state formally whether these Chr11p15.4-pter deletions occurred or not. If CRISPR-induced chromosomal deletions in HSPCs do exist, this genotoxic risk is under 0.4%. Primary cells could not tolerate haploidy over an extra-large region. As also suggested by three recent papers^17,18,30^, we previously demonstrated that the risk of chromosomal terminal deletion depends on p53 invalidation human primary fibroblastic cells^12^. HSPCs could benefit from p53 activity to prevent this risk^16^.

Further studies will be required to evaluate whether downregulation or mutations of *TP53* in HSPCs induce chromosomal terminal deletion risk. It is possible that iCN-LOH is a survival repair response to the loss of an allele in p53-proficient cells. The mechanism of CN-LOH post CRISPR-Cas9-mediated DSB is probably consistent with Break-induced replication (BIR) recently described in eukaryotic cells^31^. It involves extensive DNA synthesis from DSB to the telomere^32^ contrary to classical gene conversion with only a short kilobase-scale patch of DNA synthesis in the neighboring of the DSB^33^. DNA synthesis is imprecise during BIR process. This unusual DNA repair pathway can be responsible of frequent genetic instability^31^. A study of the mechanisms that modulate LOH induced by CRISPR-Cas9 would likely elucidate this issue.

In conclusion, our findings reveal an unexpected and unexplored form of genotoxicity that could be critical for the safety of CRISPR-Cas9 gene therapies. These induced CN-LOHs (iCN-LOHs) are easily missed by standard quality controls such as locus sequencing, FISH and even array-CGH. In parallel, chromotrypsis has been reported as an ON-target consequence of CRISPR-Cas9 genome editing^34^. The community now needs to develop technologies without subcloning to detect all ON-target rearrangements like CN-LOHs or megabase deletions for clinical purposes. Future work should be aimed not only at detecting these iCN-LOHs but also at understanding their biological roots and reasons for occurrence as well as biological roots and phenotypic consequences. DSB-free genome editing with single nickase or base editors or nuclease-based gene therapy in iPSC with safe corrected clone sorting might be answers^35–37^.

## Methods

### Cell culture

Human embryonic kidney (HEK) cell line HEK293T (ATCC, Manassas, VA, USA) was maintained in Dulbecco’s modified Eagle’s medium (DMEM), low glucose (1G.L-1), l-glutamine (1G.L-1) and pyruvate (Gibco by Lifetechnologies^TM^, Carlsbad, CA, USA) supplemented with 10% fetal bovine serum, 100 U/mL penicillin, and 100 μg/mL streptomycin (all from EurobioTM, Courtaboeuf, France). Human CD34+ HSPCs were isolated from the cord blood of healthy donors from Bagatelle Hospital, Bordeaux, France, according to the ethical Institutional review board of Bagatelle Hospital, (Maison de Santé Protestante de Bordeaux Talence FRANCE) and with the mother’s informed consent. Briefly, mononuclear cells were isolated by Ficoll gradient. CD34+ cells were purified according to the manufacturer’s instructions (Human CD34-Positive Selection kit II ref 17865 from Stem Cell Technologies) and purity was analyzed by flow cytometry using phycoerythrin-conjugated anti-CD34 antibody (clone 561 Biolegend (San Diego CA, USA) 25 μg/mL lot # B2044487 5 μL/test). Cryopreserved CD34^+^ cells were thawed and cultured in expansion medium consisting in Stem Span SFEM (Stem Cell Technologies) supplemented with Flt3-L (50 ng/mL), SCF (50 ng/mL), human TPO (50 ng/mL), human IL3 (20 ng/mL) and human IL6 (10 ng/mL) (all from Peprotech), StemRegenin 1 (SR1) (1 μM) (Stem Cell Technologies), VPA valproic acid (500 µM) (Sigma-Aldrich) (that stimulates proliferation and pluripotency of HSPC)^38^ and 100 U/mL penicillin, and 100 μg/mL streptomycin (Eurobio). Both cells were cultured at 37 °C and with 5% CO_2_ in a humidified chamber. Two days after thawing CD34^+^ cells were transfected with Cas9 RNP (see below). For cellular clonal analysis, transfected cells were isolated and sorted by BD FACS Aria 4 days after transfection. They were cultured onto 96-well plates (Corning©, Tewksbury, MA, USA) and cultured as described above (cloning efficiency 50%). SNP screening analysis was performed 3 weeks after subcloning. SNP-analyses and methylation required at least 2 months expansion (clones 34.8 and 34.15). For the #3.4 experiment, CD34^+^ cells were plated in 35-mm tissue culture dishes at 150 and 450 cells/mL with 1 mL of methylcellulose medium (Stemcell Technologies, MethoCult H4034 Optimum) and cultured at 37 °C in a humidified atmosphere containing 5% CO_2_. After 10 days, individual colonies were subsequently picked from plates and washed in PBS to remove all the methylcellulose. Cells were digested with proteinase K in lysis buffer (10 mmol/L Tris–Cl, pH 8.0, 50 mmol/L KCl, 2.5 mmol/L MgCl2, 0.5% Tween 20, 100 mg/mL proteinase K) at 56 °C for 2 h, followed by a 10-min exposure at 95 °C.

### Editing tools

HEK-293T and CD34^+^ cells were transfected by electroporation using the 4D-Nucleofector system AMAXA (Lonza®, Bale, Switzerland) with SF Cell Line 4D-Nucleofector® in association with the DG-150 program and the DO-100 program, respectively, according to the manufacturer’s instructions. To form RNP complex, Cas9 protein was complexed to crRNA:tracrRNA according to the manufacturer’s instructions. Then, complexes were incubated for 20 min at room temperature before electroporation. Cas9 protein (Alt-R® S.p. Cas9 Nuclease V3 or HiFi Cas9 V3 nuclease) and crRNA (Alt-R® CRISPR-Cas9 crRNA) were purchased from Integrated DNA Technologies (IDT, Coralville, IA, USA). Briefly, 10^6^ HEK293T cells or 150,000 primary CD34^+^ cells were nucleofected with 17 µg of nuclease-Cas9/cr/Tracr RNA complex (RNP) and 5 µM of Alt-R® Cas9 Electroporation Enhancer. Cells were then seeded onto 6-well plates (Corning^©^, Tewksbury, MA, USA) and cultured as described above.

### SNP analysis by Sanger sequencing and allele-specific quantitative PCR

We used dbSNP (NCBI, nih.gov) to screen frequent SNPs in telomeric location from the beta-globin locus. We selected and tested SNPs in *RRM1*, *CARS1 and KCNQ1* to suspect or confirm Chr11p LOH. We recovered five SNPs from SNP-array data in telomeric location from the beta-globin locus to confirm Chr11p LOH SNP-array results (Supplementary Fig. 8) and 2 framing the globin cut-site (Supplementary Fig. 9). Genomic DNA was extracted for each clone by using Nucleospin® Tissue (Macherey-Nagel, Duren, Germany) according to the manufacturer’s protocol. The genomic regions flanking SNPs were amplified by PCR (HotStarTaq Plus DNA polymerase, Qiagen®, Venlo, Netherlands) with adequate primers (Supplementary Table 5). Design of primer was made using Primer-BLAST web tool (Primer designing tool (nih.gov).). The primer annealing temperature was calculated with a Tm calculator from New England Biolabs NEB (Tm Calculator v1.13.0; https://tmcalculator.neb.com/#!/main). PCR products were purified with Nucleospin® Gel and PCR Clean-up (Macherey-Nagel) and sequenced. Alternatively, SNP genotyping was also performed by real-time quantitative PCR analysis (CFX Connect device, Biorad®) of genomic DNA with a common reverse primer and SNP allele-specific forward primers (the 3′ end nucleotide in bold is allele-specific, the underlined nucleotide in italic being a deliberate C/T mismatch introduced to enhance specificity). SNP *rs4910889* primers, *RRM1* gene (locus 11p15.4): Forward primer allele T: CCTGAGTGCCACAGTCC*C*AG**T;** Forward primer allele C: CTGAGTGCCACAGTCC*C*AG**C**; Common reverse primer: AGGCAATTCCACAGTATGGGT. SNP *rs231357* primers, *KCNQ1/KCNQ1OT1* gene (locus 11p15.5-p15.4): Forward primer allele A: ACGTTTCATAGTCAGACAAA*C*CC**A**; Forward primer allele G: CGTTTCATAGTCAGACAAA*C*CC**G**; Common reverse primer: GGGGAGGATGAAGTTAGCTGA. To validate these real-time PCR assays, PCR was performed in parallel with flanking primers encompassing the SNPs of interest, and PCR products were processed by Sanger sequencing. Quantitative allele-specific PCR analyses were quantified using the comparative MNE (Mean Normalized Expression) method,^39^.

### ICE analysis of outcome of INDELs

Genomic DNA was extracted using Nucleospin® Tissue (Macherey-Nagel, Duren, Germany) according to the manufacturer’s protocol. The genomic region flanking *HBB* exon 1, *HBG2* and *HBG1* promoter, and the 5′ breakpoint of the Corfu deletion were amplified by PCR (HotStarTaq Plus DNA polymerase, Qiagen®, Venlo, Netherlands) with adequate primers (Supplementary Table 4). PCR products were purified with Nucleospin® Gel and PCR Clean-up (Macherey-Nagel) and sequenced (LIGHTRUN, GATC Biotech, Konstanz, Germany). The Inference of CRISPR Editing (ICE) software (https://ice.synthego.com) was used to determine the percentage of indels among polyclonal populations for all protocols allowing gRNA efficiencies by ICE (Synthego). Non-transfected HEK293T and primary CD34^+^ cells are provided as control chromatogram. The limit of quantification (loq) was set at 2%.

### Cytogenetic examination of chromosomes 11 and 2

To validate the SNP loss screening method and visualize chromosomal instability, FISH was performed on interphase nuclei with probes targeting the following regions on Chr11: sub-centromeric region (XCE 11 probe, labeled in orange) (MetaSystems Probes, Altlussheim, Germany), and sub-telomeric regions (p-arm sub-telomere probes labeled in green) (Cytocell Ltd, Cambridge, UK). In parallel, Chr2 was analyzed by FISH as control. FISH was performed on interphase nuclei with probes targeting the following regions on Chr2: sub-centromeric region (XCE 2 probe, labeled in green) (MetaSystems Probes, Altlussheim, Germany), and sub-telomeric regions (q-arm sub-telomere probes labeled in red (Texas red)) (Cytocell Ltd, Cambridge, UK).

Preparations were pre-treated as indicated below. Briefly, cells were first lysed by hypotonic shock using a 2.8G/L KCl solution, then fixed using a ¾ methanol ¼ acetic acid solution and deposited onto Super Frost microscopy slides (Thermo Scientific™). The slides were successively immersed in a 2x saline-sodium citrate buffer for 10 min at 37 °C, in a 0.01% pepsin solution for 10 min at 37 °С, in a 1x phosphate-buffered saline (PBS) solution for 5 min, in a 3.7% formaldehyde solution for 5 min, and finally in a 1x PBS solution for 5 min. Then they were dehydrated by immersing them successively for 1 min in four coplins of alcohol 70°, 80°, 90°, and 100°. FISH probes and DNA were then co-denaturated according to the manufacturers’ protocols, and hybridization was performed overnight at 37 °C. The slides were then immersed successively in wash solutions and the nucleic acids were counterstained by 4,6-diamidino-2-phenylindole (DAPI). The slides were then placed under an Axio Imager-2 microscope with an epi-fluorescence source (Carl Zeiss AG, Oberkochen, Germany). The microscope was linked to the Metafer 4 software for automated image acquisition and processing (MetaSystems GmbH, Altlussheim, Germany). Magnification factor X630.

### Array-CGH and SNP array

Array-CGH was performed on 8x60k oligonucleotide microarrays, SNP+ CGH Array was performed on Genetisure Cyto 180 K CGH/SNP arrays (Agilent Technologies, CA). DNA was labeled (cyanine 3 or cyanine 5) using the Genomic DNA ULS Labeling Kit from Agilent Technologies and hybridized onto the microarrays according to the manufacturer’s instructions (Agilent).For SNP+ CGH arrays, tested DNAs were hybridized against male control DNA obtained from Agilent. Scanning of the microarrays was performed using a G2565CA scanner (Agilent). Data analysis was carried out with Agilent Technologies software, namely Feature Extraction for Cytogenomics Algorithm V5.0.1.16 for the fluorescence ratio calculation and Agilent CytoGenomics 4.0 and 5.0 to visualize chromosomal imbalances and loss of heterozygosity (LOH). Deletions and duplications in the heterozygous state were characterized by values of the log2 ratio of fluorescence intensities (cyanine 5/cyanine 3) below −0.5 and above +0.3, respectively, with the statistical algorithm ADM2 used at a threshold of 5. LOH was evaluated with the statistical algorithm ADM2 used at a threshold of 6 (Default Analysis Method v2).

### Off-target analysis

The Top-10-predicted off-target sites identified by CRISPOR software were screened to evaluate a putative off-target in Chr11 (Supplementary Table 2).

### Methylation analyses

Bisulfite conversion of 250 ng of genomic DNA was performed using the EpiTect Bisulfite Kit according to the manufacturer’s instructions (QIAGEN, Hilden, Germany). Methylation was then analyzed using real-time quantitative methylation-specific PCR (qMSP)^39^. Real-time quantitative PCR amplification conditions were 95 °C for 3 min, followed by 45 cycles at 95 °C for 30 s, 62 °C for 30 s, and 66 °C for 30 s, using a SyberGreen Master Mix (ROCHE Molecular Diagnostics), and a LC480 qPCR machine (ROCHE Molecular Diagnostics). The primers used for qMSP and BGS analyses were designed using the MethPrimer website [https://www.urogene.org/cgi-bin/methprimer/methprimer.cgi] and the OLIGO 4.0 software (Molecular Biology Insights, Inc., Colorado Springs, CO, USA). Primer sequences for the *H19* proximal promoter were: Methylated alleles: Forward: CGTTTGTTAGTAGAGTGCGTTCG, Reverse: AACCGAACTTATACTCGTCACCG; Unmethylated alleles: Forward: TATTGTTTGTTAGTAGAGTGTGTTTG, Reverse: ATTAACCAAACTTATACTCATCACCA. Primer sequences for the *H19 DMR* (differentially methylated region) were: Methylated alleles: Forward: CGGTTTTATCGTTTGGATGGTAC, Reverse: CGACGCGTAACTTAAATAACCCG; Unmethylated alleles: Forward: TTTTGGTTTTATTGTTTGGATGGTAT, Reverse: CTACAACACATAACTTAAATAACCCA. Primer sequences for the *IGF2 DMR* (differentially methylated region) were: Methylated alleles: Forward: ATTTTTTTAGGAAGTATAGTTACGTC, Reverse: ATAAAAAATACACACGAATAACCCG; Unmethylated alleles: Forward: TTTATTTTTTTAGGAAGTATAGTTATGTT, Reverse: AAATAAAAAATACACACAAATAACCCA. Primer sequences for the KCNQ1OT1 (KCNQ1 opposite strand/antisense transcript 1) DMR (differentially methylated region) were: Methylated alleles: Forward: GTTTATTATTTCGGGGTGATCGC, Reverse: CTAATCTCGAACGTAACCTAAACG; Unmethylated alleles: Forward: GTGTTTATTATTTTGGGGTGATTGT, Reverse: AACTAATCTCAAACATAACCTAAACA. Quantitative methylation-specific PCR (qMSP) analyses were quantified using the comparative MNE (mean normalized expression) method^39^, here adapted to copy numbers of unmethylated and methylated DNA alleles. Data are displayed as percentage of unmethylated and methylated alleles. PCR specificity was verified by melting curves, gel electrophoresis and Sanger sequencing analyses.

DNA from wild-type CD34^+^ cells and CRISPR-Cas9 clones 34.8 and 34.15 with iCN-LOH were analyzed by quantitative real-time methylation-specific PCR (qMSP). Wild-type peripheral blood leukocytes (PBL) from healthy donors, in vitro methylated DNA and No DNA were used as negative and positive controls. Percentages of unmethylated and methylated alleles (mean ± SEM from independent measurements) are shown.

### Gene expression analysis

Gene expression levels were determined by reverse transcription/real-time quantitative PCR (qRT-PCR), using the comparative MNE (Mean Normalized Expression) method^40^. The primers used for qRT-PCR analyses were *H19*-forward: CGTCCCTTCTGAATTTAATTTG, *H19*-reverse: ACACTCGTACTGAGACTC, IGF2- forward: GTCGCAGCCGTGGCATCGTT, *IGF2*-reverse: AGGTGTCATATTGGAAGAACTTG. To normalize the qRT-PCR data, we used the primers for the following housekeeping genes: TATA box binding protein (TBP): TBP-forward: AGTGAAGAACAGTCCAGACTG, TBP-reverse: CCAGGAAATAACTCTGGCTCAT; Glyceraldehyde-3-phosphate dehydrogenase (*GAPDH*): *GAPDH*-forward: CTGCACCACCAACTGCTTAG, *GAPDH*-reverse: AGGTCCACCACTGACACGTT; and eukaryotic translation elongation factor 1 alpha 1 (EEF1A1/EF1α): EF1α-forward: CTGGAGCCAAGTGCTAACATG, EF1α-reverse: CCGGGTTTGAGAACACCAGT. Similar results were obtained when normalizing with these three housekeeping genes. Melting curves, gel electrophoresis and sequencing analyses showed that these primers amplified only the specific fragments.

### Statistical analysis

Statistical significance was inferred when necessary. Graph Pad Prism 6 software was used for statistical analysis. Results are presented as mean ± SEM (standard error of the mean). Two-sided chi-squared tests were used to compare percentages. All comparisons are shown with black bars. Null hypothesis was rejected when *p*-value < 0.05. ns, non-significant.

### Reporting summary

Further information on research design is available in the Nature Research Reporting Summary linked to this article.

## Supplementary information

Supplementary Information

Reporting Summary

Article File - Editor summary

## Data Availability

The source data generated in this study have been deposited in the ZENODO database 10.5281/zenodo.5130579.
