# Supplementary material for: CRISPR-Cas9 globin editing can induce megabase-scale copy-neutral losses of heterozygosity in hematopoietic cells
Source: Nat Commun. 2021 Aug 13;12:4922. doi: 10.1038/s41467-021-25190-6 (PMC8363739; doi:10.1038/s41467-021-25190-6)
Supplement: Supplementary file 1 — Supplementary Information [file 41467_2021_25190_MOESM1_ESM.pdf]

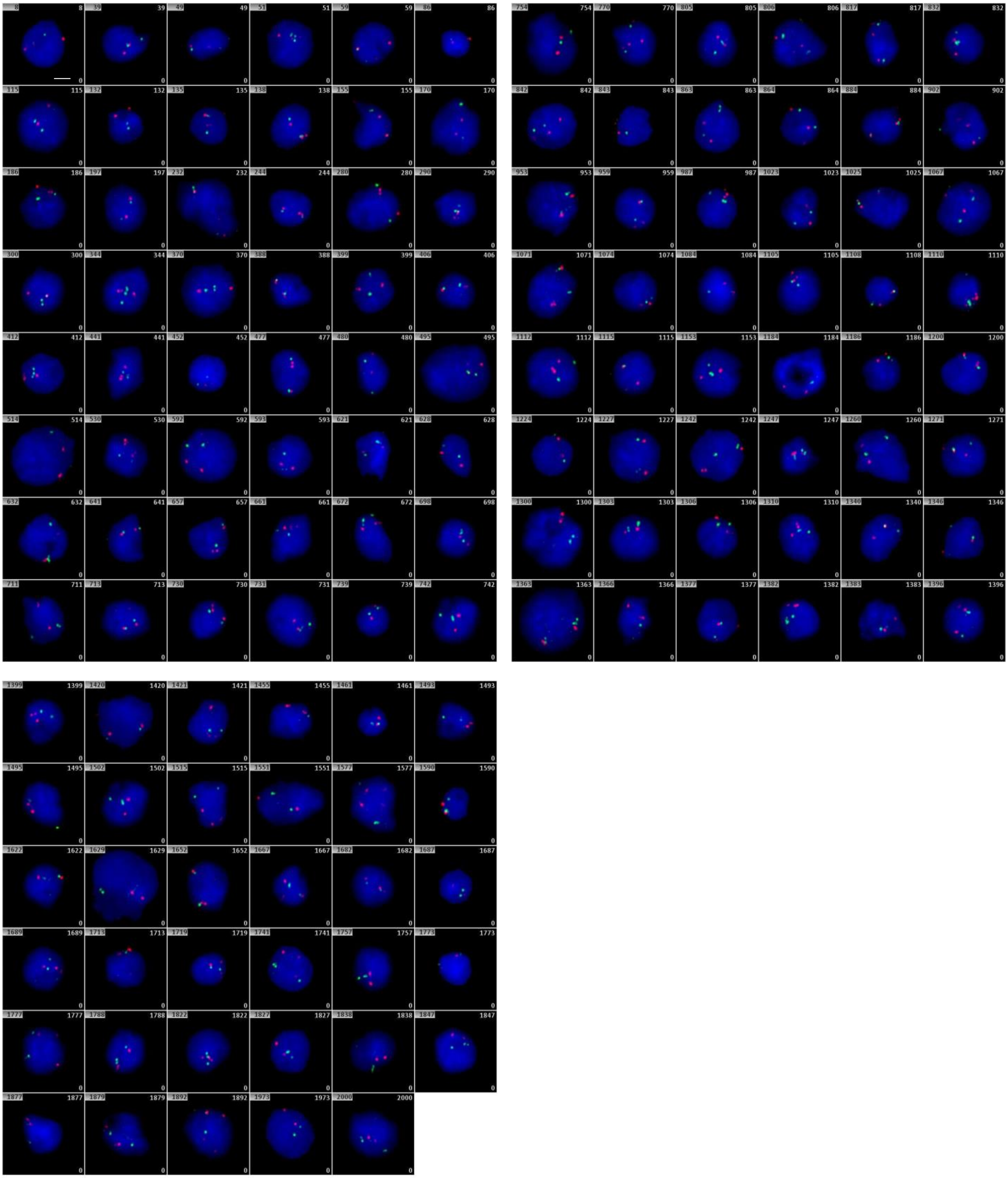

Supplementary Figure 1 Representative HEK cells FISH image gallery day 4 after protocol #1 (n=551 cells). Scale-bar: 5µm

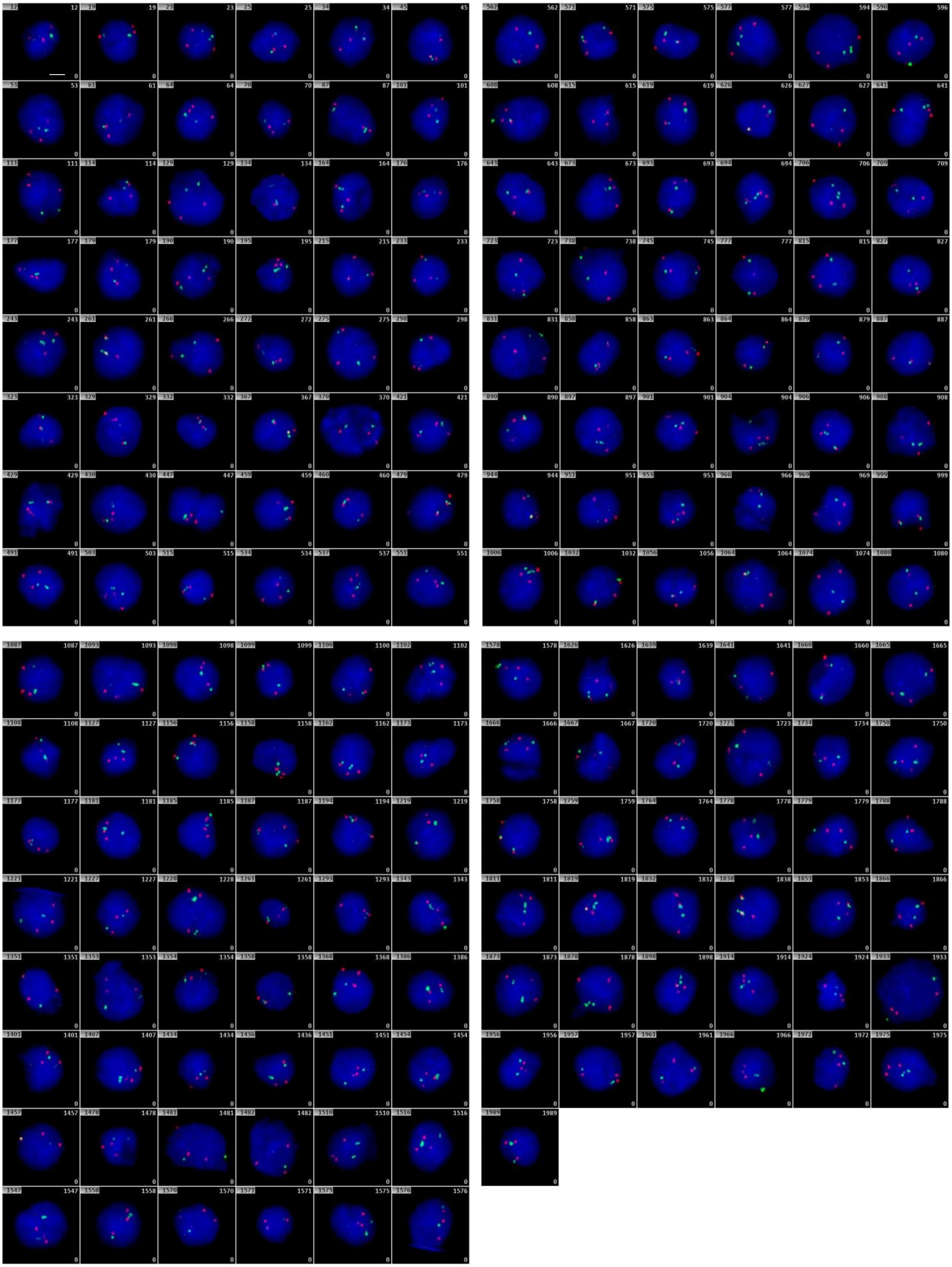

Supplementary Figure 2 Representative HEK cells FISH image gallery day 4 after protocol #2 (n=612 cells). Scale-bar: 5µm

Supplementary Fig 3

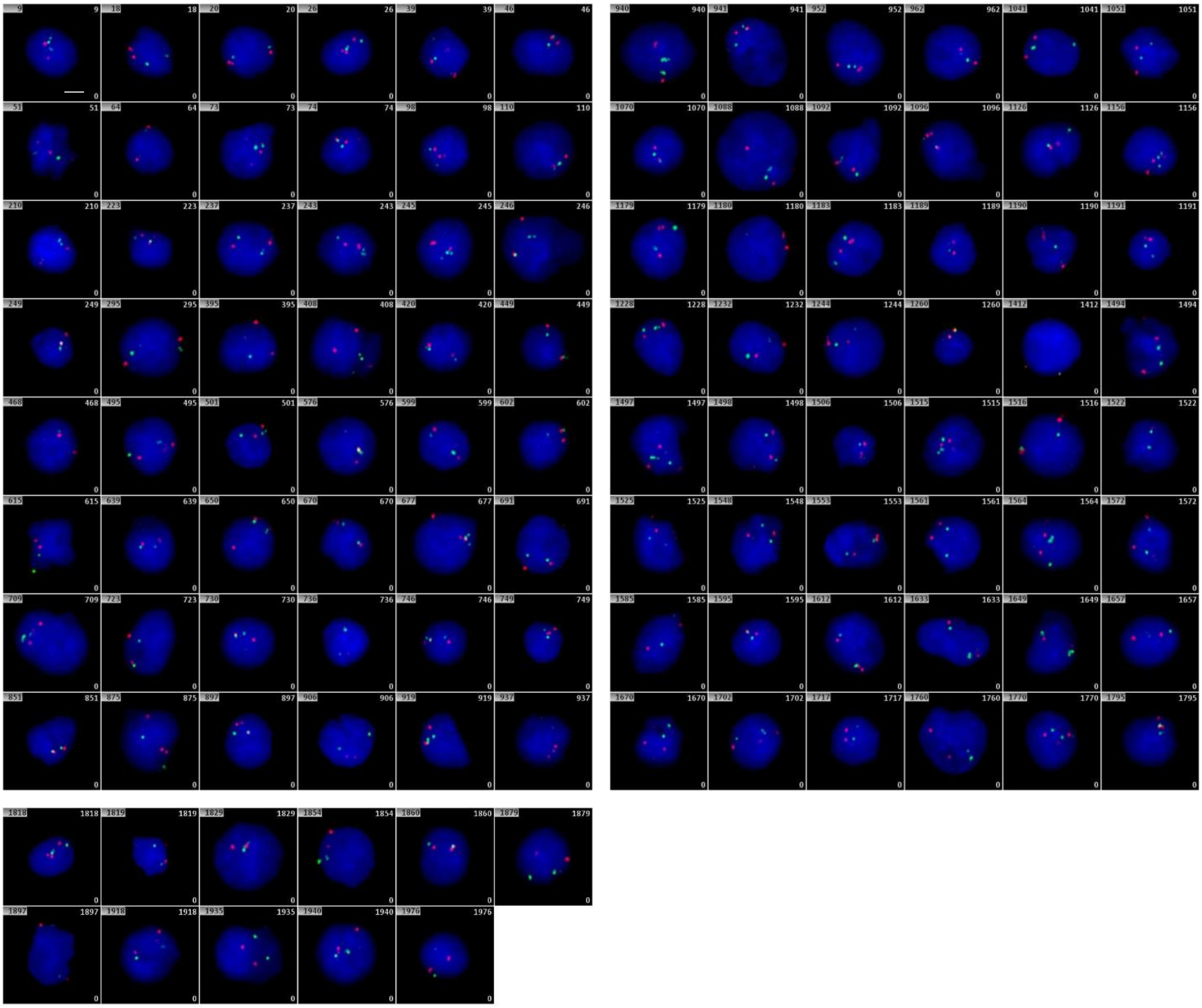

Supplementary Figure 3 Representative HEK cells FISH image gallery day 4 after protocol # 3 (n=673 cells in the experiment). Scale-bar: 5µm

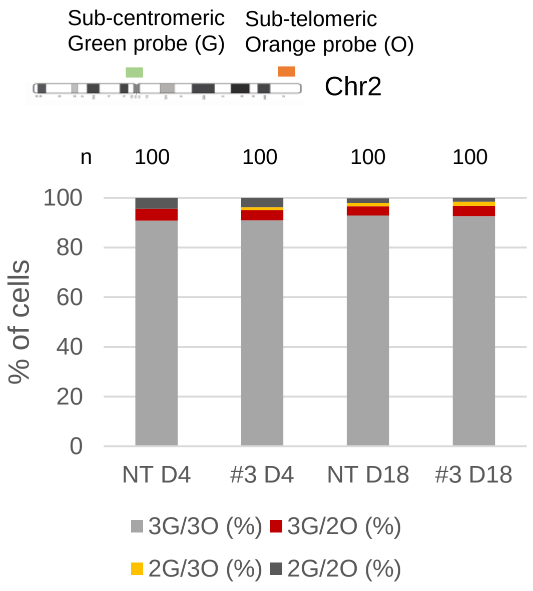

Chr 2	3G/3O (%)	3G/2O (%)	2G/3O (%)	2G/2O (%)
NT D4	90.9	4.9	0	4.3
#3 D4	90.9	4.2	1.2	3.7
NT D18	93	3.7	1.3	2
#3 D18	92.6	4.2	1.6	1.6

Supplementary Figure 4: Chr 2-FISH profile distribution of HEK cells, targeting globin genes in chr 11 at day 4 and at day 18.
After globin targeting by protocol #3 (Chr11) by nuclease, chromosome 2 integrity is analyzed at day 5 by DNA-FISH using a sub-centromeric Chr2 green probe (G) and a subtelomeric Chr2 orange probe (O). 3G/2O percentages are stable between conditions.

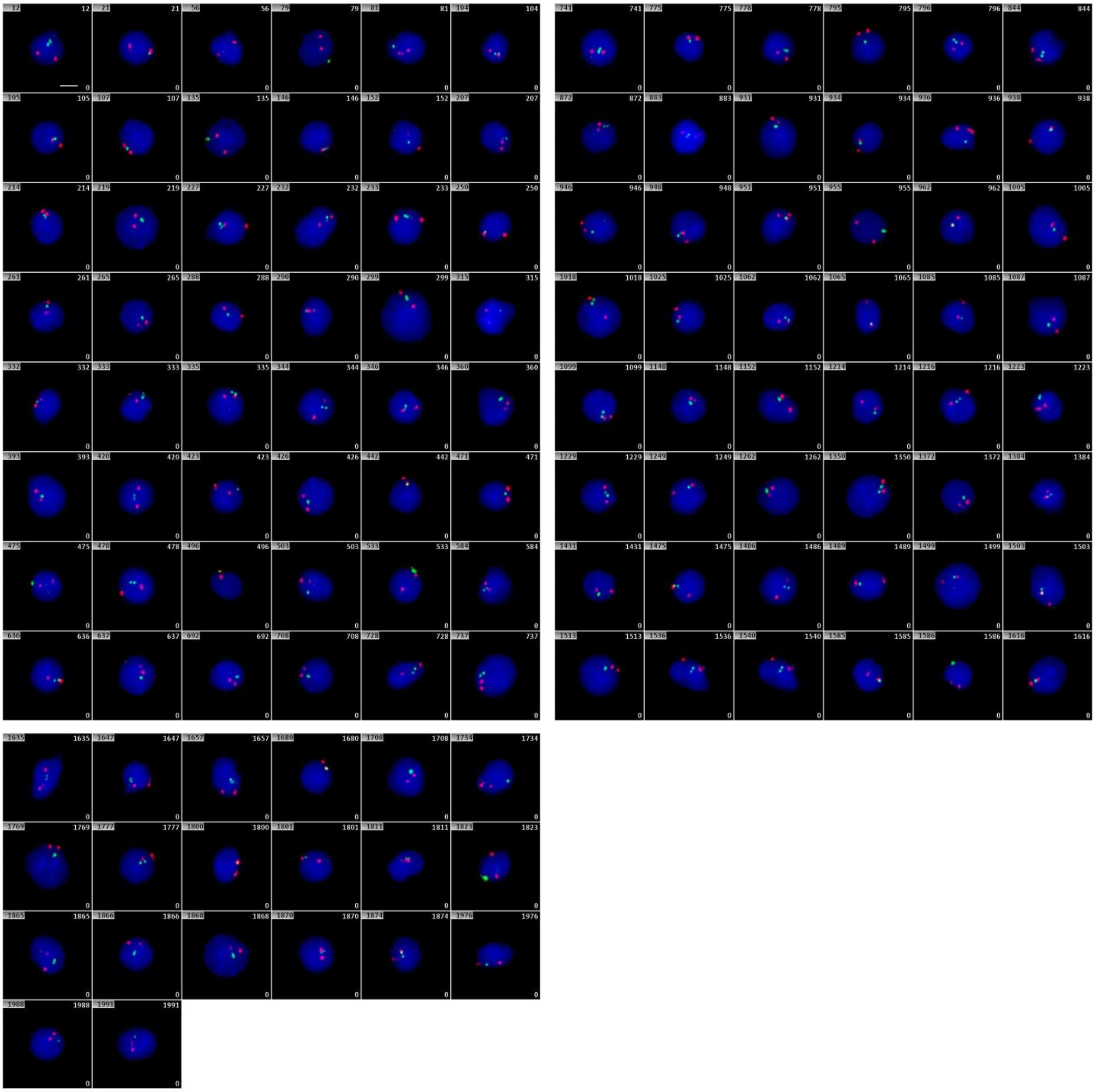

Supplementary Figure 5 Representative CD34^{+} cells FISH image gallery after protocol #1. 2 independant experiments analyzed at day 5 (n= 1100 cells). Scale-bar: 5µm

Supplementary Fig 6

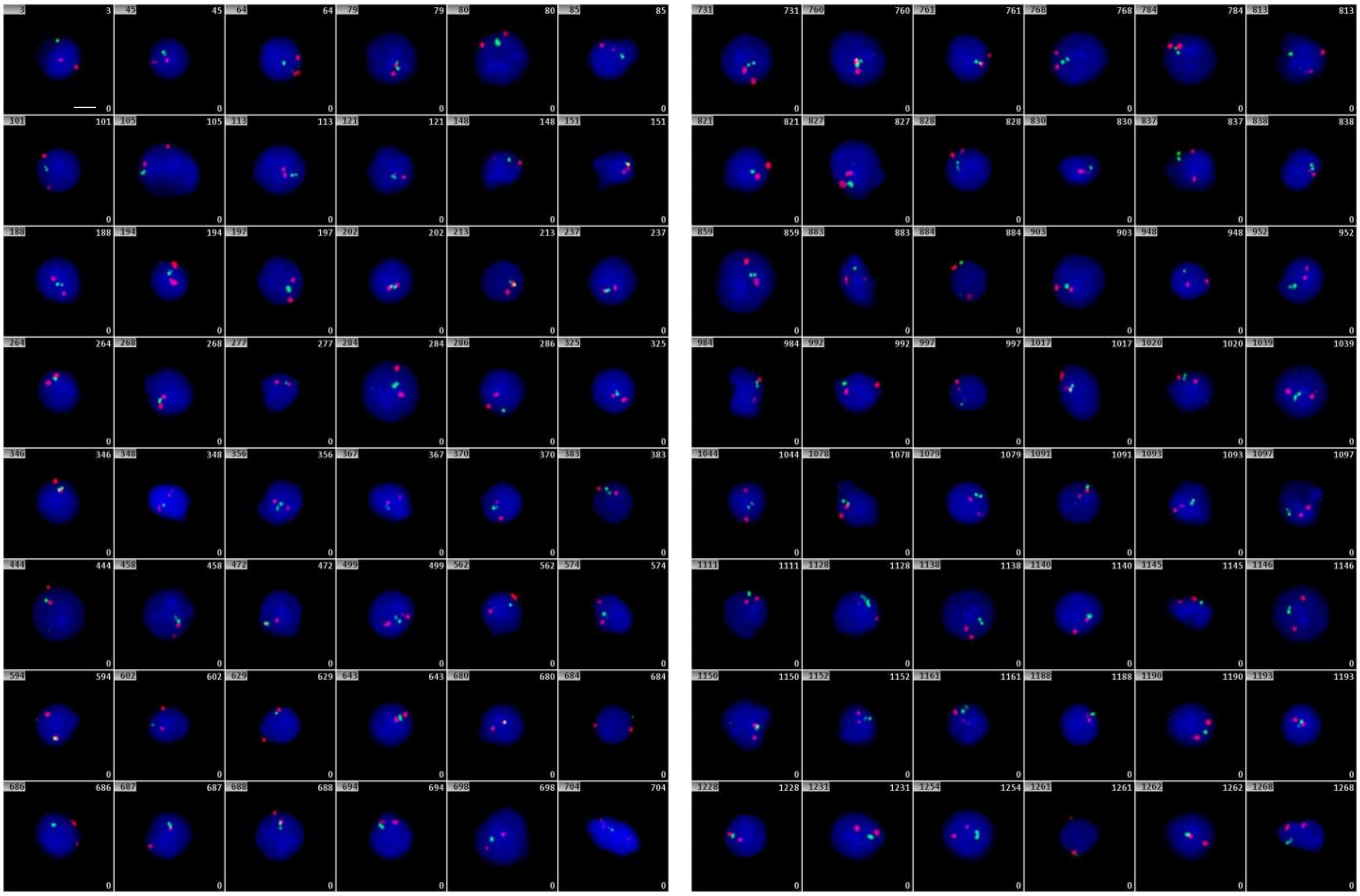

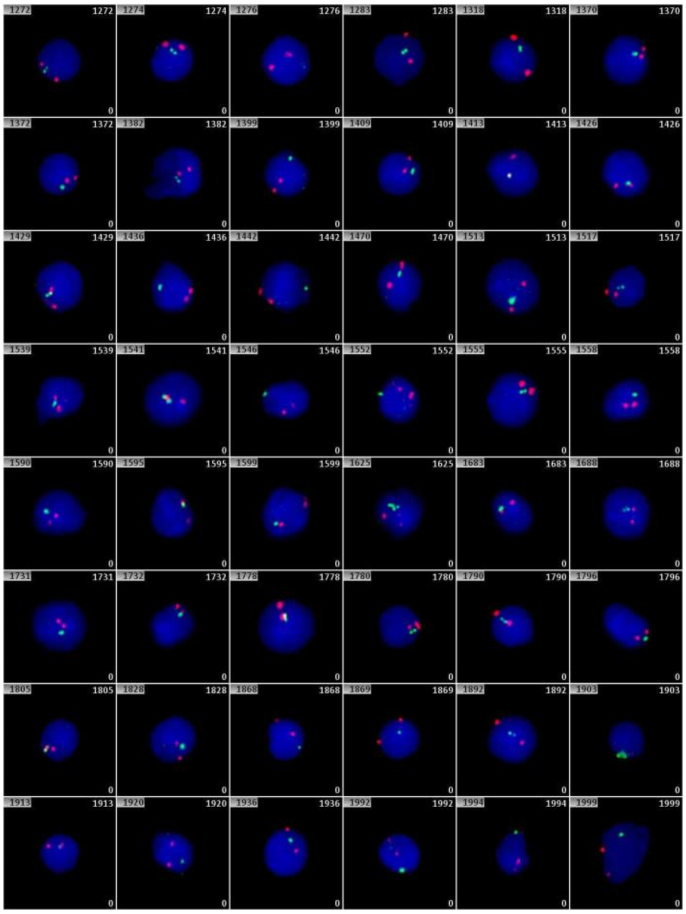

Supplementary Figure 6 Representative CD34^{+} cells FISH image gallery after protocol #3. 3 independant experiments analyzed at day 5 (n= 2189 cells). Scale-bar: 5µm.

Supplementary Fig. 7

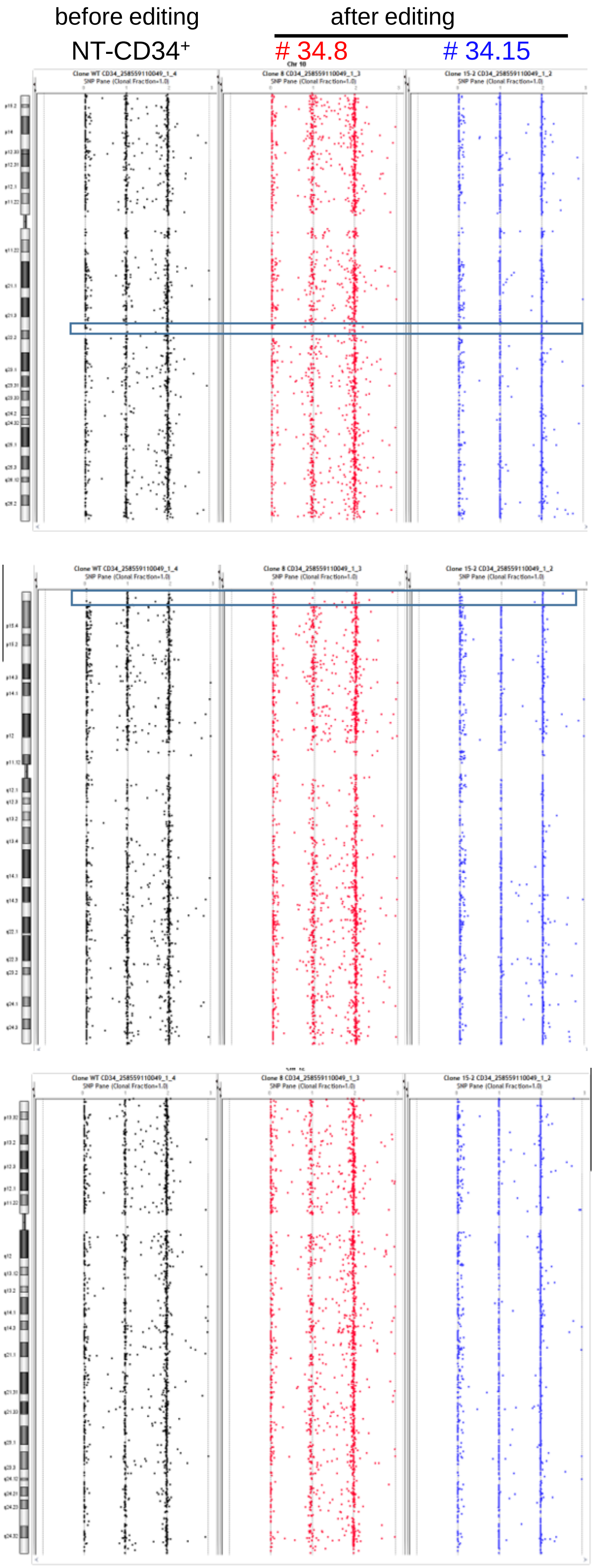

Chr10 with constitutive LOH in edited clones and in cells before editing

Chr11 with induced LOH in edited clones

Chr12 without LOH in cells before editing and edited clones

Supplementary Fig 7: SNP-array pictures of $CD34^+$ clones. Chr 10 with the single constitutive LOH, Chr11 with the single induced LOH and Chr 12, representative of all other normal chromosomes, are presented.

Supplementary Fig. 8

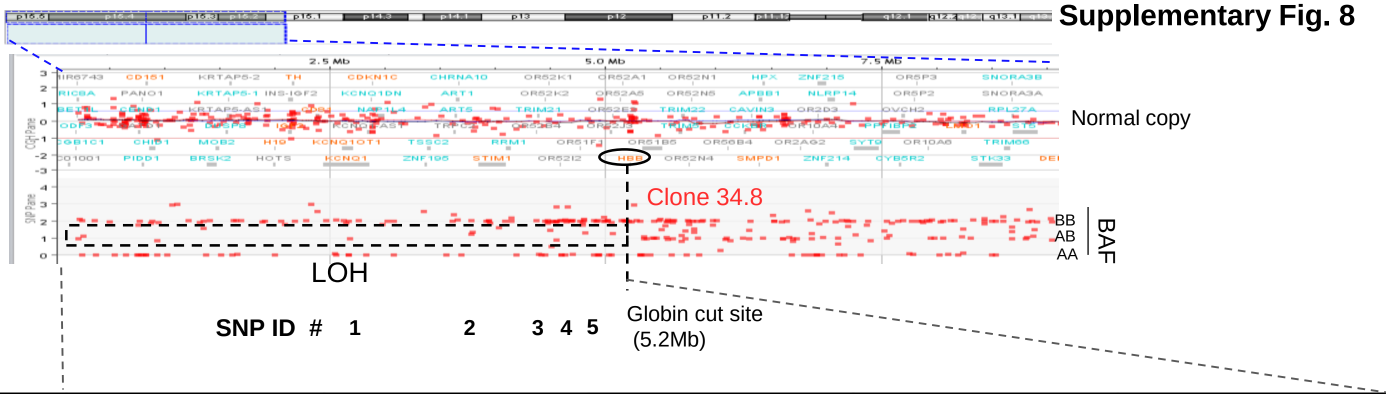

SNP ID	**#1** - rs231357	**#2** - rs7120938	**#3** - rs331512	**#4** - rs1430399	**#5** - rs10836711
Position	2.6 Mb	3.9 Mb	4.4 Mb	4.5 Mb	4.8 Mb
Fwd primer	CCCCACACTCACTGCTACAT	AGACATTTCCTCCTCCTCGG	TTTCCCCTCTGCTGCAAAAC	GCTGCCATCTACTATCCCCA	ACAGGCTTAGACTAAAGGGTGT
Rev primer	GGGGAGGATGAAGTTAGCTGA	CCAGCTGTGCCTTGTTTCAA	ACAGGCAGTGAGTCAGATTCA	GGAAAATGCCAGCCTGACAT	TGAAAGGCACAAATGAATGAGTT
Clone 34.8 (LOH pos)	A	T	G	G	G
Clone 34.15 (LOH pos)	A	T	G	G	G
Clone 1 WT (LOH neg)	A/G	C/T	A/G	A/G	A/G

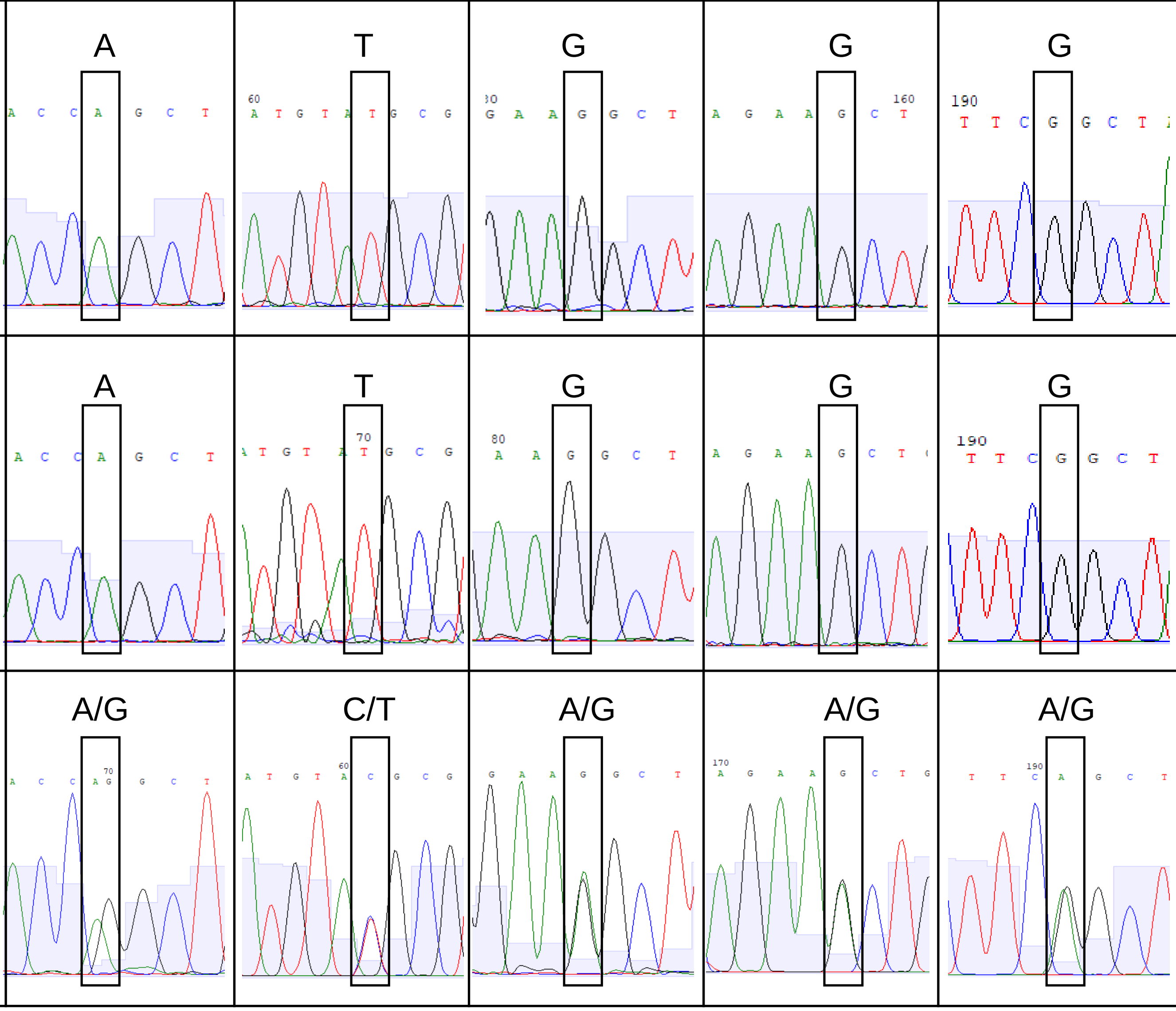

Supplemental Figure 8 : Chromosome 11p15 SNP sequencing of clones from protocols #3.1 and #3.2
SNP array analysis of clone CD34.8 showed imperfect LOH starting from *HBB* at Chr11p15.4 at 5.2 Mb towards Chr11p15.15 region. SNP sequencing was performed along the chromosome 11p15 region in clones 34.8. 34.15 and clone 1 are LOH positive and LOH negative controls respectively. Position of SNP from telomere (11p arm extremity). Source data are provided as a Source Data file

a.

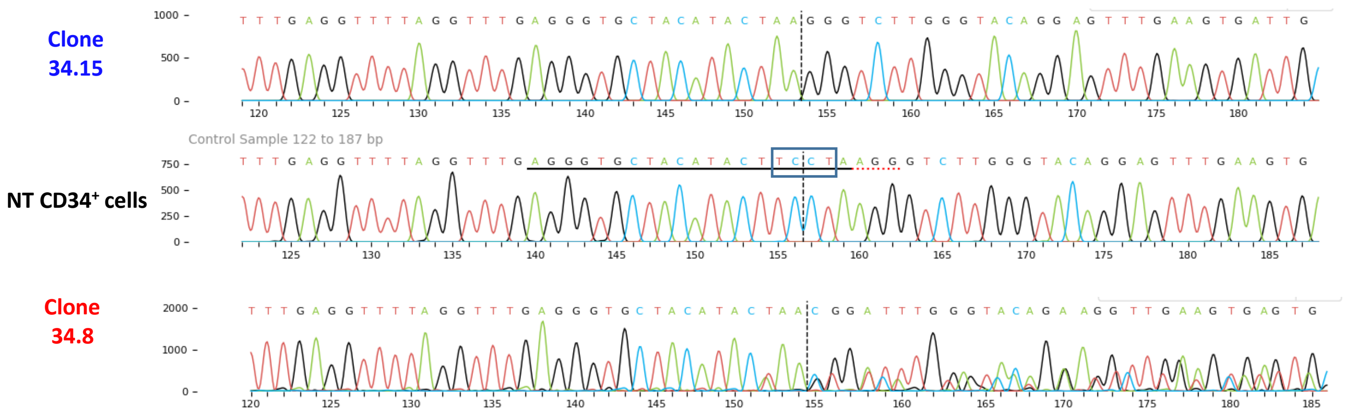

b.

Gene	*OR52E2*	*OR51B4*
SNP ID	rs1840639	rs1022241640
Primer Fwd	GCTCAAAGCTGAGGCAATCA	TGCTGTTTAGCCTCACACAAGT
Primer Rev	AGCAAGGTGCATGAAGACAGA	TCTGGGCCTCTCCCATTACT

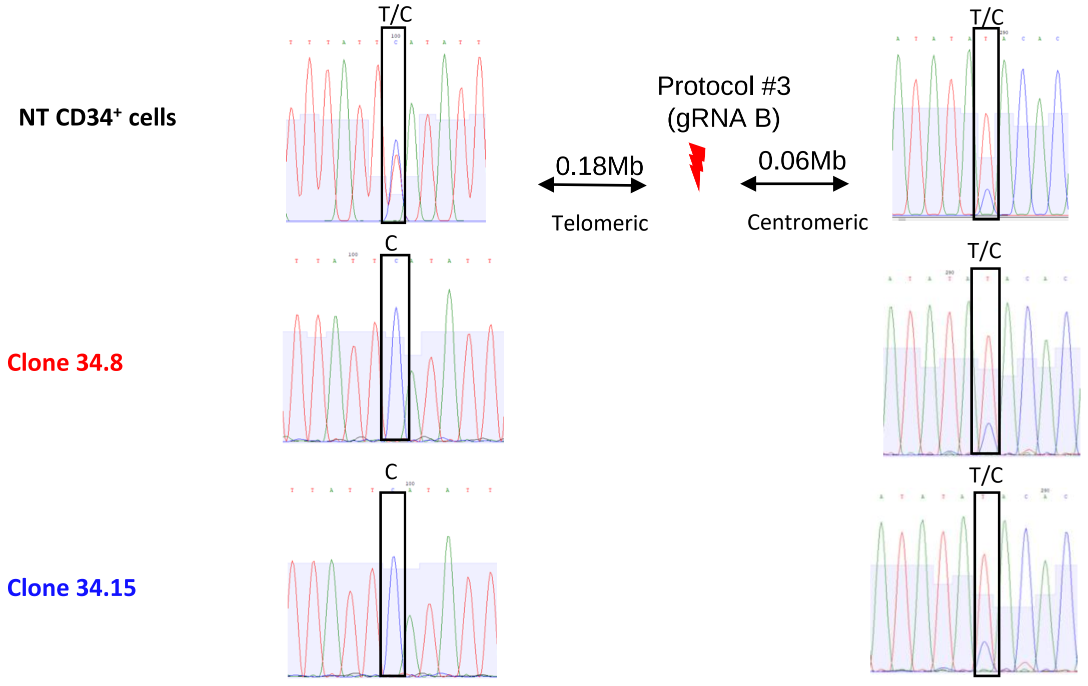

Supplementary Fig. 9

a, Sequence of the DNA breaking point site. Clone 34.15 analysis at the break point indicates a homozygous deletion of 4 base pair (CTTC 152-156). The CN-LOH is starting at the breaking point and include it. Clone 34.8 analysis revealed the presence of a heterozygous edited sequence at cut site. The CN-LOH starts probably just downstream of the breaking point. NT-CD34+ cells (non-transduced CD34+ cells).

b, Sequences of SNP framing the cutsite. In both clones, the surrounding SNPs on the telomeric become homozygous whereas the centromeric SNPs are still heterozygous. Source data are provided as a Source Data file

a

DNA methylation status:

Methylation-specific PCR products melting curve analyses

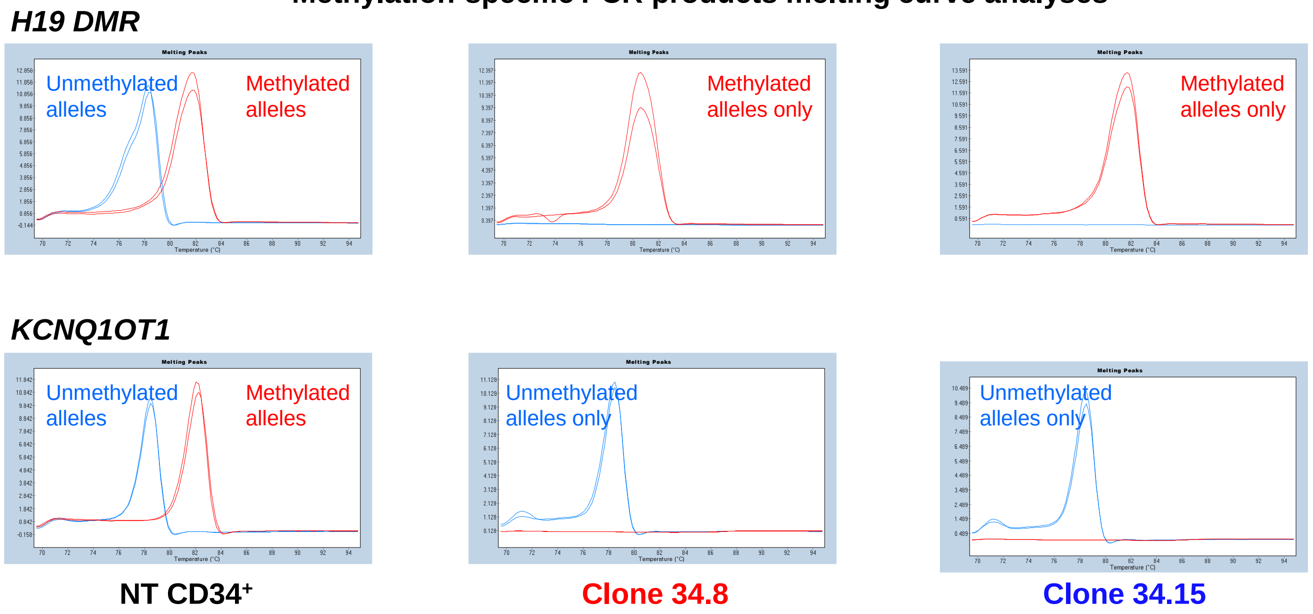

b

IGF2-AS (DMR)

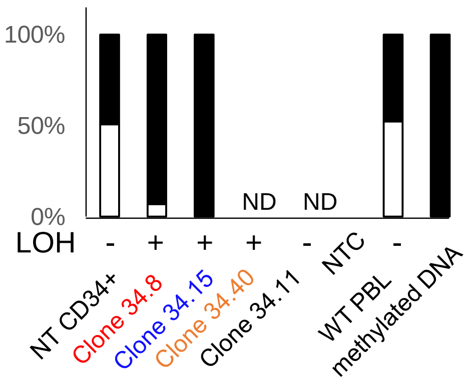

Supplementary Fig. 10: Analysis of the methylation status of the *H19/IGF2/KCNQ1OT1* imprinting centers

a, Representative examples of methylation-specific PCR products melting curves analyses for the *H19* gene differentially methylated region (*H19 DMR,* nucleotide positions analyzed here being *-2164_-2012* referring to transcription initiation site in sequence *NR_002196.2*) and for the *KCNQ1OT1* gene (*KCNQ1* opposite strand/antisense transcript 1, nucleotide positions analyzed here being -480_-457 referring to transcription initiation site in sequence NR_002728).

Graphs and melting curves analyses show that wild type CD34$^+$ cells harbor both unmethylated and methylated alleles of *H19*, *IGF2*/antisense and *KCNQ1OT1* genes, while CRISPR clones 34.8 and 34.15 with iCN-LOH harbor only methylated alleles of *H19* and *IGF2* 5'/antisense and only unmethylated alleles of *KCNQ1OT1.* These data indicate, together with those of array-CGH and SNP analysis, the occurrence of LOH resulting in duplication of the paternal Chr11p15.4-pter region.

b, Percentages of unmethylated and methylated alleles of *IGF2-AS* gene differentially methylated region (*IGF2-AS DMR)* in NT-CD34$^+$ (non-transduced parental CD34+ cells), clones 34.8, 34.15 and 34.40 with LOH and in clone 34.11 without LOH analyzed by quantitative real-time methylation-specific PCR (qMSP). WT peripheral blood leukocytes (PBL), *in vitro* methylated DNA and No DNA (NTC) were used as positive and negative controls. ND: non determined because of lack of DNA cell material.

Protocol	Clone id	SNP	SNP	FISH	CGH	LOH type
		rs2735691	rs10835611			
#1	6	C/C	C/C	2G3O	Del 11p ter	Truncation
	12	C/G	C/G	2G3O		Truncation
	28	C/G	C/G	2G3O		Truncation
	29	C/G	C/G	2G3O		Truncation
	30	C/G	C/G	3G3O	without Del	CN-LOH
	33	C/G	C/G	2G3O		Truncation
	37	C/C	C/C	2G3O		Truncation
	41	C/G	C/G	2G3O		Truncation
	43	C/C	C/C	2G3O		Truncation
	47	C/C	C/C	2G3O		Truncation
	51	C/G	C/G	2G3O		Truncation
	55	C/G	C/G	2G3O		Truncation
	63	C/G	C/G	ND		
#2	6	C/G	C/G	2G3O		Truncation
	8	C/G	C/G	2G3O	Del 11p ter	Truncation
	14	C/G	C/G	2G3O		Truncation
	23	C/G	C/G	2G3O		Truncation
	25	C/G	C/G	2G3O		Truncation
	28	C/G	C/G	2G3O		Truncation
	31	C/C	C/C	2G3O		Truncation
	34	C/G	C/G	2G3O		Truncation
	39	C/G	C/G	2G3O		Truncation
	41	C/C	C/C	3G3O	without Del	CN-LOH
#3	4	C/C	C/C	2G2O (diploid cells)		False LOH
	8	C/C	C/C	2G3O	Del 11p ter	Truncation
	9	C/G	C/G	2G3O		Truncation
	10	C/C	C/C	2G3O		Truncation
	13	C/C	C/C	2G3O		Truncation
	18	C/G	C/G	2G2O (diploid cells)		False LOH
	19	C/G	C/G	2G3O		Truncation

Supplementary Table 1: Summary table of 29 HEK clones with LOH (among 149 clones). Results of SNP sequences, FISH and representative CGH-arrays. Notably, 2 clones are diploid 2G/2O (2/149=1.3%), in accordance of their previous presence in polyclonal controls before transfection (figure 2). Among LOH HEK clones, 92.6% are truncated (telomeric chr11p loss) and 7.4% had a copy-neutral LOH (normal CGH array).

Supplementary Table 2

Protocol #1: gRNA **sequence GTAACGGCAGACTTCTCCTCAGG**

offtargetSeq	Mismatch Pos	Mismatch Count	mitOfftarget Score	cfdOfftarget Score	chr	start	strand	locusDesc
TTAAAGGAAGACTTCTCCTCAGG	*...*..*...........	3	2,543	0,3714	chr3	181783908	-	intergenic:RP11-416O18.1-RP11-416O18.2
GGAAAGGCAGACTTCTCCTTAGG	.*..*.............*	3	1,492	0,144	chr5	56047170	-	intergenic:AC022431.2-AC008940.1
GTAATGGCATATTTCTCCTCAGG	....*....*.*.......	3	1,152	0,2784	chr1	227894391	+	exon:ZNF847P
GCAAAGGCAGACTTTTCCTCAGG	.*..*........*.....	3	0,796	0,0326	chr6	11370627	-	intron:NEDD9
GGAGGGGCAGGCTTCTCCTCTGG	.*.**.....*........	4	0,794	0,2047	chr6	158896262	-	intron:TULP4
TGAGCGGCAGAGTTCTCCTCCGG	**.*.......*.......	4	0,703	0,2333	chr8	142113583	+	intergenic:RP11-128L5.1-DENND3
CTATTGGCAGATTTCTCCTCTGG	*..**......*.......	4	0,703	0,1557	chr6	38355520	-	intergenic:AL031905.1-BTBD9
TTAACGGCAGACTTCTCTTCAGA	*...............*..	2	0,689	0,0446	chr1	154268279	+	intergenic:RNU6-239P-RNU6-121P
ATAATGGAAGACTTCTTCTCAGG	*...*..*.......*...	4	0,609	0,1737	chr16	66100887	-	intergenic:RP11-356O24.1-RNA5SP428
GTATAGGGAGACTTCTTCTCTGG	...**..*.......*...	4	0,578	0,1090	chr1	66162904	-	intergenic:LEPR-RN7SL854P

Protocol #2: gRNA sequence **CTTGTCAAGGCTATTGGTCAAGG**

offtargetSeq	Mismatch Pos	Mismatch Count	mitOfftarget Score	cfdOfftarget Score	chr	start	strand	locusDesc
CTTGGCTTGGCTATTGGTCATGG	....*.**...........	3	1,584	0,12	chr15	77599510	-	intron:PEAK1
CTTTTCCAGGCTATTGGTCAAAG	...*..*............	2	0,781	0,0412	chr7	18498593	+	intron:HDAC9
CTGGAGAAGCCTATTGGTCATGG	..*.**...*.........	4	0,749	0,05	chr16	53173488	+	intron:CHD9
ATTGTCAGGCCTATTGGTCCTGG	*......*.*.........*	4	0,642	0,0666	chr12	83139785	+	intron:TMTC2
GGTATCAAGGCTATTGATCAAGG	**.*...........*...	4	0,609	0,6442	chr3	123171288	+	intergenic:ADCY5-PTPLB
TTTGTCTTTGCTATTGGTCAGGG	*.....***..........	4	0,569	0,1	chr6	8812430	+	intergenic:RP11-314C16.1-RP11-354I10.1
TTAGTATAGGCTATTGGTCAGGG	*.*..**............	4	0,556	0,2901	chr3	39556291	+	exon:MOBP
ACTGTCAGGGCTATTGGTCACAG	**.....*...........	3	0,508	0,1629	chr2	176540232	-	intergenic:AC096649.3-EXTL2P1
CTTGTCAGGGCTGTTGGTCGAGG	.......*....*......*	3	0,479	0,3657	chr5	136821768	-	intron:SPOCK1
TTTGTCTGGGCTATTGGTCCTGG	*.....**..........*	4	0,476	0,0729	chr3	821995	-	intron:AC090044.1

Protocol #3: gRNA sequence (site A) **ACCAATAGAAACTGGGCATGTGG**

offtargetSeq	Mismatch Pos	Mismatch Count	mitOfftarget Score	cfdOfftarget Score	chr	start	strand	locusDesc
AACAATAGATACTGGGCATGAGG	.*.......*..........	2	6,944	0,6417	chr8	77152103	-	intergenic:snoU13-RNU2-54P
AACAGGAGAAACTGGGCATGAGG	.*..**.............	3	1,468	0,2992	chr17	53453866	+	intergenic:RP11-515O17.3-MMD
AACTAGAGAAACTGGGCATGTGG	.*.*.*.............	3	1,468	0,2644	chr9	75644446	-	intron:ALDH1A1
AAGAGTAAAAACTGGGCATGGGG	.**.*..*...........	4	1,345	0,2618	chr4	60481397	+	intron:RP11-725D20.1
AATCTTAGAAACTGGGCATGGGG	.****..............	4	1,286	0,0641	chrX	46542070	+	intergenic:SLC9A7-YBX1P8
AAGTATAGACACTGGGCATGAGG	.***.....*.........	4	1,239	0,0771	chr4	106238408	-	intergenic:RN7SL89P-PPA2
AGCAGTAGAAACTGGGCATGAAG	.*..*..............	2	1,144	0,1298	chr3	135259720	-	intergenic:RP11-657O9.1-U8
TACAGTGGAAACTGGGCATGGGG	**..*.*............	4	0,932	0,3696	chr1	36730757	-	intron:THRAP3
AGCAACAGAAACTGGGCATCAGG	.*...*............*	3	0,902	0,2710	chr13	100850484	-	exon:RP11-340C20.3
ATCACTGGACACTGGGCATGGGG	.*..*.*..*.........	4	0,858	0,1069	chr13	111479400	-	intergenic:LINC00567-LINC00346

Protocol #3: gRNA sequence (site B) **AGGGTGCTACATACTTCCTAAGG**

offtargetSeq	Mismatch Pos	Mismatch Count	mitOfftarget Score	cfdOfftarget Score	chr	start	strand	locusDesc
AGGGTGCTCTATACTTCCTAGGG	........**.........	2	2,9373	0,537815126	chr22	25377404	+	intron:KIAA1671
CGTGAGCCACATACTTCCTATGG	*.*.*..*...........	4	1,345	0,214285714	chr3	57909466	+	intron:SLMAP
TTGGTGCTACATACTTTCTAGGG	**.............*...	3	1,290	0,296969697	chr3	74510655	+	intron:CNTN3
ATAAAGCTACATACTTCCTAAGG	.****..............	4	1,286	0,214772727	chr13	57471877	+	intergenic:RN7SKP6-PRR20A
AAGGTGCTACATACTTTCTACAG	.*.............*...	2	1,045	0,102374169	chr7	18410922	-	intron:HDAC9
AGACAGATACATACTTCCTAGGG	..***.*............	4	0,878	0,148897059	chr13	38637197	-	intron:LINC00571
ATAATGCTACCTACTTCCTAGGG	.***......*........	4	0,782	0,171818182	chr5	137065790	-	intron:KLHL3
AGGGTGTTAGATACTTCCTGTGG	......*..*.........*	3	0,779	0,241625817	chr8	21769672	-	intron:DOK2
ATAATACTACATACTTCCTAAGG	.***.*.............	4	0,778	0,429545454	chr5	64930979	-	intergenic:CTC-534A2.2-TRAPPC13
AAATTACTACATACTTCCTATGG	.***.*.............	4	0,778	0,230769231	chr22	26185619	+	intron:MYO18B

Supplementary Table 2 Predicted top10 OFF-target analyzed by CRISPOR software

Protocol #1	experiment #1		experiment #2		experiment #3	
	NT	#1	NT	#1	NT	#1
total cells (n)	523	413	396	587	434	608
2O/2G cells (%)	94.6	92.7	97.7	96.8	97	96.7
2O/2G difference	-1.9		-0.9		-0.3	
1O/2G cells (%)	0.2	0.5	0.3	0.1	1.2	0.3
1O/2G difference	0,3		-0,2		-0,9	
2O/1G cells (%)	5.2	6.8	2	3.1	1.8	3
2O/1G difference	1.6		1.1		1.2	

Protocol #3	experiment #1		experiment #2		experiment #3		experiment #4	
	NT	#3	NT	#3	NT	#3	NT	#3
total cells (n)	393	428	396	525	92	339	92	334
2O/2G cells (%)	94.9	90.7	97.7	96.2	97.8	95.2	97.8	92.2
2O/2G difference	-4.2		-1.5		-2.6		-5.6	
1O/2G cells (%)	1.8	0.9	0.3	0.2	0	0.3	0	0.3
1O/2G difference	-0,9		-0,1		0,3		0.3	
2O/1G cells (%)	3.3	8.4	2	3.6	2.2	4.5	2.2	7.4
2O/1G difference	5.1		1.6		2.3		5.2	

Supplementary Table 3

Polyclonal Chr11 DNA-FISH analysis at D18 of edited cells with protocols #1 and #3. percentages in non-transfected cells as control (NT, Non-Transfected) and edited cells with protocols #1 and #3 (n=7).

INDELS		
Primer sequence		Protocol #
HBB Fw	AGTCAGGGCAGAGCCATCTA	1
HBB Rv	CTGTCTCCACATGCCCAGTT	
HBG Fw	ACGGCTGACAAAAGAAGTCCT	2
HBG Rv	AGCCTTGTCCTCCTCTGTGA	
G1A Fw	AGGCCATCACTAAAGGCACC	3
G1A Rv	AGTCAGGGCAGAGCCATCTA	
G1B Fw	AGCACCGCCTATCTATGTGC	
G1B Rv	GGAAACTGGATGCAGAGACCA	

Supplementary Table S5

SNP screening			
Primer sequence SNP screening		Target SNP	Target cell
RRM1 F1	GCACAGGACCTGACATGAAC	rs2735691 rs10835611	HEK293T
RRM1 R1	TGCTCTTTGGTCAGCTAAAATGT		
RRM1 F2	CCTGCCTGATGAGGAAAGATG	rs4910888 rs4910889	CD34$^+$
RRM1 R2	AGGCAATTCCACAGTATGGGT		
CARS F7	GGAGGTGCCGTACATCTCAC	rs369461 rs399565	CD34$^+$
CARS R7	GACCTTGGCACTGCAACTAAA		
KCNQ1 F	CCCCACACTCACTGCTACAT	rs231357	CD34$^+$
KCNQ1 R	GGGGAGGATGAAGTTAGCTGA		
