# Supplementary material for: CRISPR-Cas9 globin editing can induce megabase-scale copy-neutral losses of heterozygosity in hematopoietic cells
Source: Nat Commun. 2021 Aug 13;12:4922. doi: 10.1038/s41467-021-25190-6 (PMC8363739; doi:10.1038/s41467-021-25190-6)
Supplement: Supplementary file 2 — Reporting Summary [file 41467_2021_25190_MOESM2_ESM.pdf]

nature research

Corresponding author(s): Francois MOREAU-GAUDRY

Last updated by author(s): Jul 22, 2021

Reporting Summary

Nature Research wishes to improve the reproducibility of the work that we publish. This form provides structure for consistency and transparency in reporting. For further information on Nature Research policies, see our Editorial Policies and the Editorial Policy Checklist.

Statistics

For all statistical analyses, confirm that the following items are present in the figure legend, table legend, main text, or Methods section.

n/a	Confirmed	
☐	☒	The exact sample size (*n*) for each experimental group/condition, given as a discrete number and unit of measurement
☐	☒	A statement on whether measurements were taken from distinct samples or whether the same sample was measured repeatedly
☐	☒	The statistical test(s) used AND whether they are one- or two-sided<br>*Only common tests should be described solely by name; describe more complex techniques in the Methods section.*
☒	☐	A description of all covariates tested
☒	☐	A description of any assumptions or corrections, such as tests of normality and adjustment for multiple comparisons
☐	☒	A full description of the statistical parameters including central tendency (e.g. means) or other basic estimates (e.g. regression coefficient) AND variation (e.g. standard deviation) or associated estimates of uncertainty (e.g. confidence intervals)
☐	☒	For null hypothesis testing, the test statistic (e.g. *F*, *t*, *r*) with confidence intervals, effect sizes, degrees of freedom and *P* value noted<br>*Give P values as exact values whenever suitable.*
☒	☐	For Bayesian analysis, information on the choice of priors and Markov chain Monte Carlo settings
☒	☐	For hierarchical and complex designs, identification of the appropriate level for tests and full reporting of outcomes
☒	☐	Estimates of effect sizes (e.g. Cohen's *d*, Pearson's *r*), indicating how they were calculated

Our web collection on statistics for biologists contains articles on many of the points above.

Software and code

Policy information about availability of computer code

Data collection	no software for data collection.
Data analysis	Graph Pad Prism 6 software, presence of indels analysed with ICE (interference of CRISPR Edits) software (ice.synthego.com) CHOPCHOP v2 and algorithm ADM2 Agilent CytoGenomics Edition 5.0.1.6, Database od Single Nucleotide polymorphism NCBI (nih.gov); Annealing temperature with Tm calculator from New England Biolabs v1.13.0. off-target sites identified by CRISPOR software. Primer-BLAST (NCBI C++ toolkit

For manuscripts utilizing custom algorithms or software that are central to the research but not yet described in published literature, software must be made available to editors and reviewers. We strongly encourage code deposition in a community repository (e.g. GitHub). See the Nature Research guidelines for submitting code & software for further information.

Data

Policy information about availability of data

All manuscripts must include a data availability statement. This statement should provide the following information, where applicable:
- Accession codes, unique identifiers, or web links for publicly available datasets
- A list of figures that have associated raw data
- A description of any restrictions on data availability

All data generated or analysed during this study are included in this published article (supplementary information files and doi http://doi.org/10.5281/zenodo.5121400).

April 2020

Field-specific reporting

Please select the one below that is the best fit for your research. If you are not sure, read the appropriate sections before making your selection.

☒ Life sciences ☐ Behavioural & social sciences ☐ Ecological, evolutionary & environmental sciences

For a reference copy of the document with all sections, see nature.com/documents/nr-reporting-summary-flat.pdf

Life sciences study design

All studies must disclose on these points even when the disclosure is negative.

Sample size	No sample size calculation was performed.
Data exclusions	no data was excluded
Replication	At least 3 independent experiments. All attempts at replication were successful. DNA Methylation analyses and Q-RT-PCR were run in duplicate (fig 5b and 5d).
Randomization	no randomization was performed.
Blinding	FISH analysis is in blind. The authors conducted behavior experiments without blinding only when data were automatically recorded.

Reporting for specific materials, systems and methods

We require information from authors about some types of materials, experimental systems and methods used in many studies. Here, indicate whether each material, system or method listed is relevant to your study. If you are not sure if a list item applies to your research, read the appropriate section before selecting a response.

Materials & experimental systems

n/a	Involved in the study	
☐	☒	Antibodies
☐	☒	Eukaryotic cell lines
☒	☐	Palaeontology and archaeology
☒	☐	Animals and other organisms
☐	☒	Human research participants
☒	☐	Clinical data
☒	☐	Dual use research of concern

Methods

n/a	Involved in the study	
☒	☐	ChIP-seq
☐	☒	Flow cytometry
☒	☐	MRI-based neuroimaging

Antibodies

Antibodies used	Anti-CD34+ PE clone #561, Biolegend lot # B2044487 5 microl/test 100 microl staining volume
Validation	Biolegend: Each lot of this antibody is quality control tested by immunofluorescent staining with flow cytometric analysis.

Eukaryotic cell lines

Policy information about cell lines

Cell line source(s)	HEK293T cell from ATCC<br>Human CD34+ HSPCs were isolated from the cord blood of three healthy donors from Bagatelle Hospital, Bordeaux, France, according to ethical standards and with the mother's informed consent
Authentication	Authenticated by ATCC : Quality control specifications by STR profiling
Mycoplasma contamination	PCR-free mycoplasma
Commonly misidentified lines (See ICLAC register)	no commonly misidentified cell line were used in this study

Human research participants

Policy information about studies involving human research participants

Population characteristics	Human CD34+ HSPCs were isolated from the cord blood of healthy donors from Bagatelle Hospital, Bordeaux, France,
Recruitment	Recruitment according to ethical standards and with the mother's informed consent
Ethics oversight	Cord blood (CB) sample were obtained after maternal informed consent according to procedures approved by the Bagatelle Hospital Ethics Board

Note that full information on the approval of the study protocol must also be provided in the manuscript.

Flow Cytometry

Plots

Confirm that:

- [x] The axis labels state the marker and fluorochrome used (e.g. CD4-FITC).
- [x] The axis scales are clearly visible. Include numbers along axes only for bottom left plot of group (a 'group' is an analysis of identical markers).
- [x] All plots are contour plots with outliers or pseudocolor plots.
- [x] A numerical value for number of cells or percentage (with statistics) is provided.

Methodology

Sample preparation	Human CD34+ HSPCs were isolated from cord blood after Ficoll isolation by magnetic cell sorting
Instrument	BD Accuri FACS
Software	BD accuri C6 plus software
Cell population abundance	Puritiy is validated using CD34+ percentage (> 90%)
Gating strategy	FSC/SSC gates of the starting population

- [ ] Tick this box to confirm that a figure exemplifying the gating strategy is provided in the Supplementary Information.
